# Hirshfeld atom refinement based on projector augmented wave densities with periodic boundary conditions

**DOI:** 10.1107/S2052252522001385

**Published:** 2022-02-26

**Authors:** Paul Niklas Ruth, Regine Herbst-Irmer, Dietmar Stalke

**Affiliations:** a Institut für Anorganische Chemie, Georg-August-Universität Göttingen, Tammannstraße 4, Göttingen, Lower Saxony 37077, Germany

**Keywords:** Hirshfeld atom refinement, aspherical atomic form factors, periodic calculations, hydrogen positions, projector augmented waves

## Abstract

Hirshfeld atom refinement (HAR) is an X-ray diffraction refinement method that, in numerous publications, has been shown to give H-atom bond lengths in close agreement with neutron diffraction derived values. Presented here is a first evaluation of an approach using densities derived from projector augmented wave (PAW) densities with three-dimensional periodic boundary conditions for HAR. The results show an improvement over refinements that neglect the crystal environment or treat it classically, while being on a par with non-periodic approximations for treating the solid-state environment quantum mechanically. A suite of functionals were evaluated for this purpose, showing that the SCAN and revSCAN functionals are most suited to these types of calculation.

## Abstract 2

Deriving the atomic form factors from Hirshfeld-partitioned periodic projector augmented wave calculations shows a great benefit for H-atom bond lengths and a smaller benefit for H-atom atomic displacement parameters.

## 1. Introduction

Jayatilaka & Dittrich (2008 ▸) proposed a new method to obtain aspherical form factors for use in X-ray structure refinement: they suggested using the Fourier transform of atomic densities, which are obtained by Hirshfeld stockholder partitioning (Hirshfeld, 1971 ▸) of theoretically derived densities. This approach was further developed by Capelli *et al.* (2014 ▸) to use an iterative approach. Compared with the ubiquitous independent atom model, the authors were able to show a benefit for the experimental determination of *X*—H bond lengths, and several studies have been conducted to establish this fact further. A comprehensive study showed a strong correlation of *X*—H bond lengths from neutron diffraction and Hirshfeld atom refinement (HAR) in 25 different classes of *X*—H bonding motifs, derived from HAR of 81 structures in total (Woińska *et al.*, 2016 ▸). Direct comparisons on a structure-to-structure basis also verified this fact using densities derived from Hartree–Fock calculations (Fugel *et al.*, 2018 ▸) and density functional theory (DFT) (Sanjuan-Szklarz *et al.*, 2020 ▸; Woińska *et al.*, 2014 ▸). The accuracy of H-atom bond lengths and displacement parameters has also been evaluated for metal hydride bonds in a recent publication (Woińska *et al.*, 2021 ▸).

Since that initial work, the focus has shifted from validation to tapping new sources for Hirshfeld partitioned densities. *NoSpherA2* (Kleemiss *et al.*, 2021 ▸), as implemented in *OLEX* (Dolomanov *et al.*, 2009 ▸), has broadened the scope of crystallographic questions by enabling the refinement of phenomena like disorder. Additionally, it provides an easy-to-use interface to *ORCA* (Neese, 2018 ▸), *PySCF* (Sun *et al.*, 2020 ▸) and *TONTO* (Jayatilaka & Grimwood, 2003 ▸). The software *lamaGOET* (Malaspina *et al.*, 2021*a* ▸) offers an interface from *GAUSSIAN* (Frisch *et al.*, 2009 ▸) to *TONTO*, as well as a way to use extremely localized molecular orbitals (ELMOs) from the ELMOdb (Meyer & Genoni, 2018 ▸). The ELMO approach enables an extremely fast HAR, while still providing a benefit over independent atom model refinements (Malaspina *et al.*, 2019 ▸). The higher-level theoretical methods MP2 and CCSD have also been evaluated recently (Wieduwilt *et al.*, 2020 ▸). While the crystal field was neglected in that study of l-alanine, the derived structure factors did show a higher agreement compared with Hartee–Fock and DFT calculations with the BLYP functional, while CCSD also showed an improvement over B3LYP derived densities. However, in comparison with cheaper computational methods, systematic improvements to the agreement of *X*—H bond lengths with neutron values were not observed.

In addition to the investigations exploring the influence of density calculation methods, a recent study also investigated the effect of density partitioning methods on the H-atom parameters (Chodkiewicz *et al.*, 2020 ▸). A slight improvement for the usage of iterative stockholder methods over HAR was observed. However, the authors themselves note that more research in this new and interesting direction is needed.

A more sophisticated treatment of the crystal environment via ELMO embedding (Wieduwilt *et al.*, 2021 ▸) showed impressive agreements of H-atom bond lengths with neutron diffraction derived values for xylitol. The authors also suggested a useful hierarchy of crystal environment descriptions which they arranged in the common form of a Jacob’s Ladder. On the first step they put the neglect of the crystal environment. The following step is the classical description of the environment with the help of charges and/or multipoles. Currently, the highest step in Hirshfeld methods is the quantum description of the environment. Wieduwilt and co-workers realized this quantum description by embedding the molecule into a region described by an ELMO basis set.

Here we want to contribute results from a different approach calculating densities in molecules in such a high-level description of the solid state, namely using densities derived from projector augmented waves (PAWs) (Blöchl, 1994 ▸) in periodic DFT calculations. There is a single previous study (Wall, 2016 ▸) that used densities calculated with the *Vienna ab initio Simulation Package* (*VASP*) (Kresse & Furthmüller, 1996 ▸) for the description of urea. The study used calculations from different geometries, but the refinement was not executed iteratively. To the best of our knowledge, this source of density has not been investigated any further since that initial case study.

The projector augmented wave method (Blöchl, 1994 ▸) is a frozen-core all-electron method, shown to be closely related to the pseudopotential method (Kresse & Joubert, 1999 ▸). It enables the reconstruction of the all-electron wavefunction from the pseudo wavefunctions via a linear transformation. This means we retain the good convergence properties with respect to the number of plane waves or real-space grid points, but are still able to partition a complete density to obtain atomic form factors. There are several mature packages that can be used for these calculations, such as *QUANTUM ESPRESSO* (Giannozzi *et al.*, 2017 ▸), *ABINIT* (Gonze *et al.*, 2020 ▸) or *GPAW* (Hjorth Larsen *et al.*, 2017 ▸). We decided to use the third option, as it is *Python*-based and therefore enabled us to build the crystallographic part of the HAR with the well established *NumPy* (Harris *et al.*, 2020 ▸) and *SciPy* (Virtanen *et al.*, 2020 ▸) libraries, using the automatic gradient determination of *JAX* (Bradbury *et al.*, 2018 ▸). Our custom Python library programmed for this purpose has been named ‘X-ray diffraction data Hirshfeld atom refinement in Python’ or *XHARPy*.

## 2. Methodology

### 2.1. Calculation of aspherical form factors

If not noted differently below, the aspherical form factors were calculated from densities obtained by real-space grid projector augmented wave calculations in *GPAW* (Mortensen *et al.*, 2005 ▸; Enkovaara *et al.*, 2010 ▸) using the atomic simulation environment (*ASE*) (Hjorth Larsen *et al.*, 2017 ▸) and interpolated to a finer grid using the built-in routine. The valence parts of the atomic form factors were obtained by partitioning the valence densities and using the fast Fourier transform (FFT) algorithm as implemented in *NumPy* (Harris *et al.*, 2020 ▸) before finally shifting the phase to obtain the atom-centred values. Meanwhile, the core part of the atomic form factor was calculated once at the beginning of each refinement by numerical Fourier–Bessel transform of the spherical frozen-core density on an exponential grid with 2^19^ + 1 points. This also means that the core density was not included in the Hirshfeld partitioning, but was instead assigned completely to the respective atom. Accordingly, Hirshfeld weights were also determined without the frozen-core density.

In total we evaluated 12 functionals within *GPAW*: PW (Perdew & Wang, 1992 ▸), BLYP (Becke, 1988 ▸; Lee *et al.*, 1988 ▸), PW91 (Perdew *et al.*, 1992 ▸), PBE (Perdew *et al.*, 1996 ▸), revPBE (Zhang & Yang, 1998 ▸), RPBE (Hammer *et al.*, 1999 ▸), TPSS (Tao *et al.*, 2003 ▸; Sun *et al.*, 2015 ▸; Perdew *et al.*, 2004 ▸), SCAN (Sun *et al.*, 2015 ▸), revSCAN (Mezei *et al.*, 2018 ▸), vdW-DF (Dion *et al.*, 2004 ▸, 2005 ▸), vdW-DF2 (Lee *et al.*, 2010 ▸) and BEEF-vdW (Wellendorff *et al.*, 2012 ▸). Due to convergence problems in the periodic DFT calculations in *GPAW*, no hybrid functionals were included.

For comparison, we also performed Hirshfeld atom refinements using *NoSpherA2* (Kleemiss *et al.*, 2021 ▸) as implemented in *OLEX* (Dolomanov *et al.*, 2009 ▸), using *ORCA* (Neese, 2018 ▸) as the engine for the density calculation of isolated molecules and *TONTO* (Jayatilaka & Grimwood, 2003 ▸) for the calculation of atomic form factors derived from densities embedded in a cluster of Hirshfeld charges. Calculations in *NoSpherA2* employed the B3LYP functional with the def2-TZVPP basis set. Further information about the settings used, as well as the calculated fragments, can be found in the supporting information in Section S3.2. In order to assure comparability between refinements, we did not refine a weighting scheme.

### 2.2. Structure refinement

Structure refinement for the PAW-HAR method was implemented in a custom Python package using *NumPy* and *JAX* (Bradbury *et al.*, 2018 ▸) for array calculations and automatic gradient generation, and the *BFGS* (Broyden, 1970 ▸; Fletcher, 1970 ▸; Goldfarb, 1970 ▸; Shanno, 1970 ▸) implementation of *scipy.optimize* for optimization. All refinements were done against *wR*_2_(*F* ^2^) with *w* = 1/σ^2^. Calculation of the phase was done in the established way (Coppens, 2010 ▸). This is despite using FFT of the rectangular density grid for the atomic form factor calculations, where a different calculation has been suggested in the literature (Wall, 2016 ▸). For our reasoning see Section S1 in the supporting information.

We validated the correct performance of the refinement in the *XHARPy* package on the independent atom model against *SHELXL* (Sheldrick, 2015 ▸) for one structure. The results can be found in Section S2.

All Hirshfeld atom refinements were started with values derived from *SHELXL* independent atom refinements, where H atoms bound to C atoms were placed using a riding model, while H atoms that were bound to heteroatoms were refined freely with isotropic displacement parameters. Visualization of X-ray structures was done with *ShelXle* (Hübschle *et al.*, 2011 ▸).

### 2.3. Quality indicators for comparison

To compare the performance of different HAR approaches, we benchmarked to both neutron data and *wR*_2_(*F* ^2^). The agreement with neutron diffraction results was evaluated for bond lengths to H atoms and for the anisotropic displacement parameters of H atoms. Distances were compared directly. However, as differences in absorption or extinction, and small deviations in temperature, between neutron and X-ray diffraction experiments can influence anisotropic displacement parameters, the neutron displacement parameters were scaled using equation (1) (Blessing, 1995 ▸) implemented in our own *Python* script:

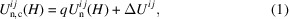

where *q* and Δ*U^ij^* were determined by a least-squares fit to the non-H atoms and were determined for each HAR refinement individually. The factor *q* represents scaling due to differences in measurement temperature between the neutron and the X-ray experiments. 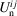 is the atomic displacement parameter determined by neutron diffraction and 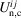 is the corrected neutron displacement parameter used for further comparisons.

The following quality indicators were used to estimate the relative performance of different functionals within the PAW-HAR method, as well as the performance compared with comparison and reference calculations:

(i) *wR*_2_(*F* ^2^) is a scaled least-squares agreement factor. As such, a lower *wR*_2_(*F* ^2^) should indicate a higher precision in determined values. However, due to possible systematic deviations, the *wR*_2_(*F* ^2^) does not determine accuracy alone. This means that, in method development, a comparison of H-atom bond lengths and displacement parameters with values derived from other sources is inevitable.

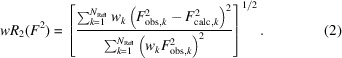




(ii) Δ*r* and |Δ*r*|. Currently, the main application of HAR is the determination of H-atom positions: Therefore, the difference in *X*—H bond lengths from reference neutron values is the central criterion to evaluate the quality of any endeavour. The difference is simply calculated as

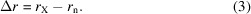




To see immediately the performance in our figures, we used the absolute value of this difference |Δ*r*| for most of our figures. This means that a lower value always indicates an improved agreement.

(iii) Δ*U_ij_* and |Δ*U_ij_* |. Additionally, a high agreement in the calculated displacement parameters is also desirable. As such, the difference from the scaled reference neutron values is calculated. Again, it is simply calculated as

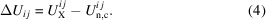




Consistent with the distances, we used the absolute value of this difference |Δ*U_ij_* | for most of our figures, so that a lower value always indicates an improved agreement.

(iv) *S*_12_. To compare the deviation between the probability distributions described by the H-atom anisotropic displacement parameters from different sources (in our case refinements from X-ray and neutron data), the *S*_12_ value has been proposed (Whitten & Spackman, 2006 ▸). If 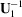 and 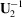 are the inverses of displacement matrices in the Cartesian convention from the respective sources, it is calculated as

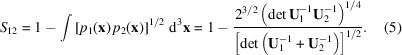

Usually, this equation has an additional factor of 100. As we give the values in percent, our numerical values are equivalent.

### 2.4. Included datasets

We chose a variety of different datasets for use in the development of our method. We cannot rely on the least-squares agreement alone, as error compensation might lead to erroneous conclusions. The ultimate goal is to derive positions that are more accurate. To evaluate this target, both a neutron and an X-ray dataset need to be available. Additionally, the X-ray dataset should have a measured resolution of at least 0.6 Å. The scaling of the independent atom refinement should be independent of the resolution and no outliers should be visible.

Additionally, we tried to include datasets which were already subjected to a benchmark HAR. This enables us not only to compare with *NoSpherA2* results, but also with state-of-the-art results from different groups. Finally, we tried to include structures that contain H atoms engaged in hydrogen bonds and C—H bonds. This permits us to aggregate the H-atom quality descriptors in order to investigate the different performance of functionals for the two binding motifs.

**Figure 1:**
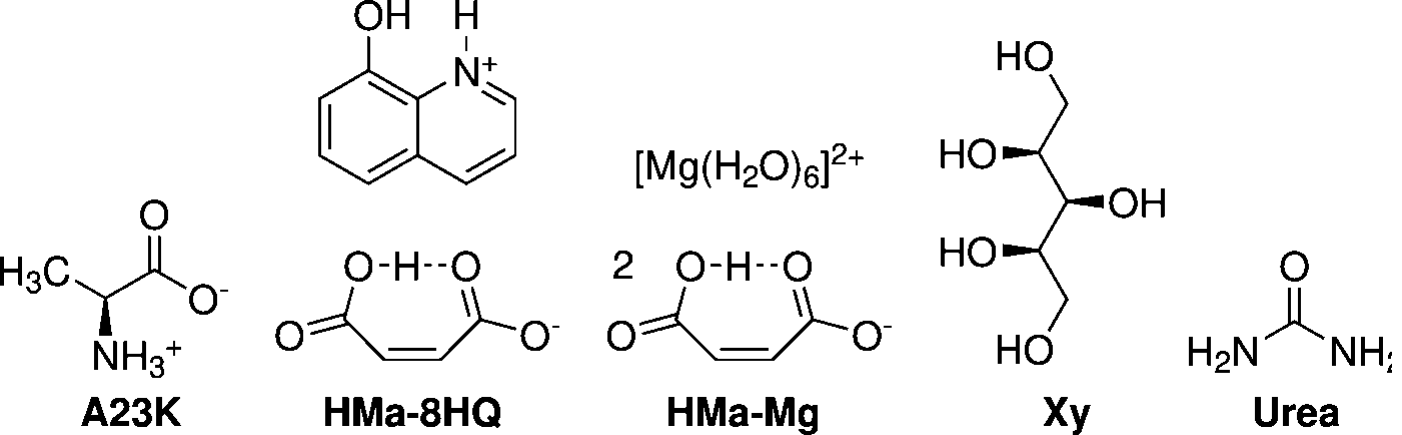
Lewis diagrams of all included structures.

The following datasets were included in our investigation (Fig. 1 ▸).

(i) l-Alanine, referred to herein as **A23K**. The initial high-resolution X-ray data at 23 K were originally collected in 1988 on a 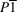 diffractometer (Destro *et al.*, 1988 ▸). These data were later complemented by the corresponding neutron diffraction dataset (Malaspina *et al.*, 2019 ▸). l-Alanine has comparatively few atoms and a small unit cell, while not having atoms on special positions. It features H atoms that are involved in classical hydrogen bonds (H1–H3) as well as H atoms that are located in C—H bonds (H4–H7). This makes it ideally suited to exploring different theoretical approaches in a reasonable amount of time. We can compare with a recent refinement that uses densities calculated by the CCSD method (Wieduwilt *et al.*, 2020 ▸).

(ii) 8-Hydroxyquinone hydrogen maleate (denoted **HMa-8HQ**) and (iii) hexaaquamagnesium hydrogen maleate (**HMa-Mg**). Recently, high-resolution X-ray diffraction data for a group of different hydrogen maleate salts have been published (Malaspina *et al.*, 2020 ▸). The corresponding neutron data were published earlier (Malaspina *et al.*, 2017 ▸). Two of these structures have been revisited in a more recent publication (Malaspina *et al.*, 2021*b* ▸). Both structures contain an H atom that is coordinated between the carb­oxy­lic acid entities of the maleate (H1). Two additional H atoms are bound to the C atoms of the maleate (H2 and H3). **HMa-8HQ** and **HMa-Mg** differ in their counter-ion. **HMa-8HQ** contains an organic heteroaromatic counter-ion. It is bound by hydrogen bonds ( atoms H4 and H5), but contains additional C-bound H atoms (atoms H6–H11). **HMa-Mg** comprises a hexaaquamagnesium dication counter-ion, exhibiting additional hydrogen bonds (atoms H4–H9). We can compare the re-refinement of the two structures from 2021. From the available refinements we chose the calculations done with the B3PW91/def2-TZVP functional.

(iv) Xylitol (denoted **Xy**). The high-resolution X-ray dataset (Madsen *et al.*, 2004 ▸) of xylitol was published shortly after the initial neutron diffraction data (Madsen *et al.*, 2003 ▸). Again, the structure contains numerous H atoms involved in hydrogen bonds (atoms H11–H15) while the remaining H atoms are located in C—H bonds. We will compare it with a recent refinement which showed impressive results calculating the density within two clusters (Wieduwilt *et al.*, 2021 ▸). The molecule itself was described with a cc-pVTZ basis set. The immediate surroundings were described by a cluster still calculated with DFT using ELMOs as the basis set. The outermost cluster shell was built using a classical description. The functional employed was B3LYP.

(v) **Urea**. The initial neutron data at 123 K were collected in 1984 (Swaminathan *et al.*, 1984 ▸), while the corresponding high-resolution X-ray dataset is more recent (Birkedal *et al.*, 2004 ▸). Using this dataset, we specifically wanted to test the performance on atoms on special positions. Both independent H atoms are involved in hydrogen bonds. Comparison can be done with a high-level B3LYP calculation that uses cluster dipoles around a quantum-mechanically calculated cluster to simulate the crystal field (Chodkiewicz *et al.*, 2020 ▸).

### 2.5. Visualization of results

In order to visualize the distribution of the given quality indicators for the investigated H atoms, this work relies heavily on box-whisker plots. In a box-whisker plot the edges of the box represent the 25th and 75th percentiles. The central line within the box marks the median value. The whisker on the left-hand side extends to the smallest point within the 25th percentile minus 1.5 times the interquartile range. The right-hand-side whisker is defined as the largest point with the 75th percentile plus 1.5 times the interquartile range. Values outside this range are potential outliers and are indicated separately by a glyph (usually a filled circle). For a more in-depth description see the work of Krzywinski & Altman (2014 ▸).

Improvement can be seen in two ways, by a smaller median disagreement (as seen by the white central line) or by a narrower distribution of the values, as indicated by the width of the boxes within the plots and, to some degree, the width of the whiskers, provided that the number of outliers does not increase at the same time.

In most cases there is an additional *x* axis on top for |Δ*r*| and |Δ*U_ij_* |. This axis is divided by the mean estimated standard deviation of the neutron distances and atomic displacement parameters for the relevant structure. This is not as accurate as dividing all deviations individually (as done in Section 3.5) but should give an estimate of how the differences compare with the neutron refinement uncertainties at little additional cost in space.

## 3. Results

### 3.1. Evaluation of real-space and *k*-point grid sizes

We used a real-space grid as the basis for the calculated wavefunctions. Just as the basis set has an influence on the result of linear combination of atomic orbitals (LCAO) calculations, a finer grid spacing should improve the quality of the calculation at the cost of computational resources. As the grid always spans the complete unit cell, the cost for a given spacing is also highly dependent on the unit-cell size, which means that for periodic PAW calculations structures with centrings are sometimes limited in how fine a grid spacing can be calculated for a given amount of system memory.

Additionally, periodic calculations integrate the first Brillouin zone via a mesh of *k*-points. A generally accepted method for calculating suitable meshes is the concept of Monkhorst–Pack grids (Monkhorst & Pack, 1976 ▸), which was also the method of choice within this work. In general, stronger interactions to neighbouring unit cells, and smaller unit cells in general, require a finer integration of the first Brillouin zone and therefore a larger *k*-point grid. However, the cost of the calculation scales linearly with the number of *k*-points.

**Figure 2:**
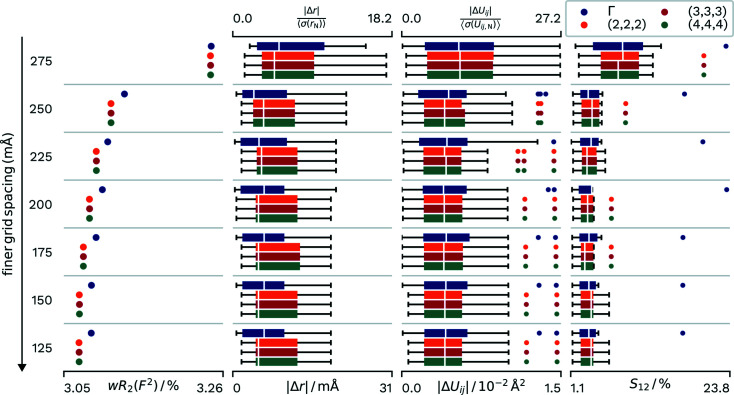
Differences in *wR*_2_(*F* ^2^) and agreement with neutron values for different real-space grid spacings and different samplings of the reciprocal space. Distributions of parameters are displayed as box-whisker plots. Different horizontal groups correspond to the real-space grid spacing for the wavefunction calculation, which is interpolated once for the density evaluation and interpolated another time for the calculation of atomic form factors via FFT. The four differently coloured sets within this group correspond to the different *k*-point samplings.

In our first step we want to evaluate the influence of the sizes of these two grids on the results of HAR. As our dataset we chose **A23K**, because it is small enough to allow more elaborate calculations, but also contains H atoms located on general positions. All calculations were done using the SCAN functional that has proved to yield the best results in preliminary evaluations. The results are depicted in Fig. 2 ▸. The given grid spacings correspond to the wavefunction grid. For density evaluations the grid spacing was half the size. For the calculation of the structure factors the density was interpolated to a grid spacing that is one quarter of the original grid, via *GPAW*’s own interpolation method. *k*-points were shifted to include the Γ point.

As a result, all quality indicators show converging improvement with finer grid spacing in real space. The *wR*_2_(*F* ^2^) improves from 3.24% at a grid spacing of 0.275 Å to 3.09% at grid spacings of both 0.125 and 0.150 Å. Increasing the *k*-points from Γ-point sampling to (2,2,2) improved the *wR*_2_(*F* ^2^) slightly to 3.07%, with no further improvement with larger sampling.

The Δ*r* values also showed a converging improvement with finer real-space grid spacing, with convergence occurring below 0.2 Å. The introduction of additional *k*-points into the calculations actually led to a slight decrease in the agreement between calculated and neutron-derived *X*—H distances, with the mean absolute difference at a real-space grid spacing of 0.125 Å increasing from 0.007 to 0.009 Å from a Γ-point sampling to a sampling of (2,2,2). Again, a further increase in *k*-points did not yield a difference in results.

The anisotropic displacement parameters improved with both a finer real-space and *k*-point grid spacing. The *S*_12_ at the Γ point converged at a real-space grid spacing of 0.175 Å. The introduction of a (2,2,2) *k*-point grid improved the agreement in displacement parameters, with the mean *S*_12_ falling for the H atoms.

To summarize, an increase in real-space grid points does always benefit the desired quantities, even though there are diminishing benefits. The agreement of distances with the neutron values actually decreases slightly for l-alanine when the number of *k*-points is increased, while the agreement in displacement parameters increases. For this molecular compound with hydrogen bonds, a finer *k*-point spacing than (2,2,2) is not necessary, because of the relatively flat band dispersion in molecular crystals.

### 3.2. Evaluation of different functionals for the calculation of hexaaquamagnesium hydrogen maleate (HMa-Mg)

We have tested the performance of PAW-HAR with a large number of functionals for all the structures evaluated in this work. The overall trends are similar. Therefore, for the sake of clarity, we limit the evaluation of functionals here to one structure, namely hexaaquamagnesium hydrogen maleate (**HMa-Mg**). The interested reader will find a summary of all other evaluations in the supporting information in Section S5. Details of the parameters for the theoretical calculations can be found in Section S3.

**Figure 3:**
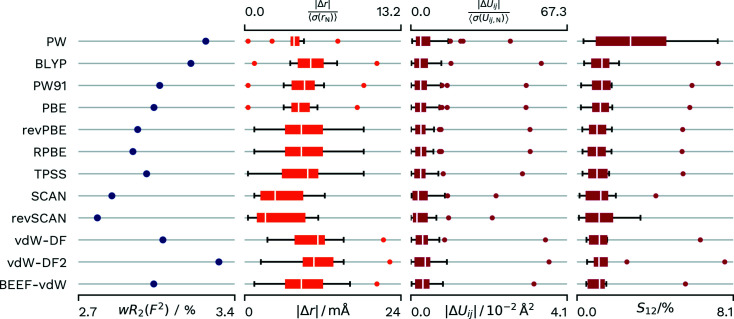
Differences in *wR*_2_(*F* ^2^) and agreement with neutron values for different functionals applied to the **HMa-Mg** dataset. Distributions of parameters are displayed as box-whisker plots. All calculations are done at the Γ point. Grid spacings: wavefunction 0.15 Å, density 0.075 Å, atomic form factor 0.0375 Å.

The hierarchy of functionals can clearly be seen in the *wR*_2_(*F* ^2^) depicted in Fig. 3 ▸. The poorest performance is shown by the vdW-DF2 functional, closely followed by the PW functional. This position is unsurprising as it only uses the LDA approximation. On the GGA level the newest evaluated functional (RPBE) also achieved the best fit to the measured intensities within its class. On the meta-GGA level the TPSS shows a lower agreement than the SCAN functional, which itself shows a slightly lower agreement than the re­parametrized revSCAN functional. BEEF-vdW exhibits the highest agreement within the evaluated van der Waals functionals, with vdW-DF2 performing poorly in general.

Surprisingly, the PW functional performs well for the evaluation of *X*—H distances. This clear an effect is unique to this dataset. However, PW usually shows an advantage over BLYP and a distance performance similar to GGA functionals. SCAN and revSCAN show the best overall performance. In this dataset revSCAN has only a small lead. The determined Δ*U_ij_* values show one outlier for all functionals. This corresponds to the *U*_11_ value of the H atom located within the hydrogen maleate molecule, where the direction of the disagreement is approximately located along the intra­molecular O—H⋯O hydrogen-bond interaction.

In consequence, we decided to use the SCAN functional as reference for further comparisons in the following evaluations.

### 3.3. Comparison of results with other methods and reference calculations

In this section, we compare our results with different reference calculations in order to investigate the performance in comparison with different density descriptions. We ordered the structures according to the positions of the reference structures on the Jacob’s Ladder proposed for HAR (Wieduwilt *et al.*, 2021 ▸). The l-alanine structure described with CCSD represents the limit of what can be reached on the first rung, which stands for no crystal field description. The reference for the two hydrogen maleate structures employed a cluster of classical charges and is therefore located on the second rung. Finally, the xylitol and urea references both used embedding in a quantum mechanically treated cluster for their HAR and are therefore located on the third rung, the quantum-mechanical description of the surroundings. We want to demonstrate that PAW-HAR also belongs to that level. A depiction of the described Jacob’s Ladder can be found in the supporting information, Fig. S3.

#### 3.3.1. l-Alanine at 23K

**Figure 4:**
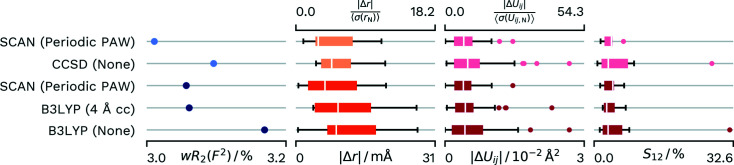
Differences in *wR*_2_(*F* ^2^) and agreement with neutron values for different density sources for HAR of **A23**. Distributions of parameters are displayed as box-whisker plots. SCAN (Periodic PAW) was conducted with our script and *GPAW*. The CCSD (None) values are from the reference calculation from the literature (Wieduwilt *et al.*, 2020 ▸). B3LYP (4 Å cc) uses 4 Å of cluster charges and was refined with *NoSpherA2*/*TONTO*. B3LYP (None) was calculated with *NoSpherA2*/*ORCA* without any approximation of the crystal environment. Lighter coloured datasets have an *F*/σ(*F*) cut-off of 3 as applied in the reference.

The refinement against the PAW-derived atomic form factors and the comparison refinements in *NoSpherA2* all result in a difference electron density that does not contain systematic features. The comparison refinement (Wieduwilt *et al.*, 2020 ▸) does use high-level theory (CCSD). At this high level it does not take the crystal environment into account. As an *F*/σ(*F*) cut off of 3 was set in the refinement, we decided to do two PAW refinements for the dataset, one that enables the best comparison by using the same data and one where we used the full data. A summary of the performance of the different methods can be found in Fig. 4 ▸. The *wR*_2_(*F* ^2^) comparison, however, confirms that the crystal environment is needed for the appropriate density description. Both the *TONTO* refinement with cluster charges and the periodic calculation show an almost identical *wR*_2_(*F* ^2^) value. Interestingly, a larger cluster radius in the cluster charge calculation led to a worse performance, while with both radii the calculation did not fully converge in 20 cycles. Meanwhile, both the comparison calculation based on a B3LYP calculation in *ORCA* and the calculation using atomic form factors from a CCSD calculation show a higher *wR*_2_(*F* ^2^) value compared with our corresponding PAW-HAR refinements.

Compared with the cluster charge calculation, the PAW-derived values show a better agreement for the distances and a slightly better agreement for the displacement parameters. Both the reference CCSD calculation (Wieduwilt *et al.*, 2020 ▸) and the single-molecule B3LYP calculation demonstrate the need for a description of the crystal field for an optimal calculation of the atomic form factors and the calculation of more accurate *X*—H distances and H-atom atomic displacement parameters.

#### 3.3.2. 8-Hydroxy­quinone hydrogen maleate and hexa­aqua­magnesium hydrogen maleate at 15 K

**Figure 5:**
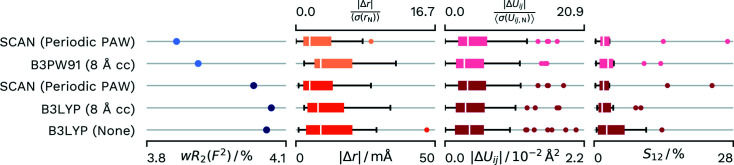
Differences in *wR*_2_(*F* ^2^) and agreement with neutron values for different density sources for HAR of **HMa-8HQ**. Distributions of parameters are displayed as box-whisker plots. SCAN (Periodic PAW) was conducted with our script and *GPAW*. The B3PW91 (8 Å cc) values are from the reference calculation from the literature (Malaspina *et al.*, 2021*b* ▸) and used 8 Å of cluster charges for the crystal approximation. B3LYP (8 Å cc) uses 8 Å of cluster charges and was refined with *NoSpherA2*/*TONTO*. B3LYP (None) was calculated with *NoSpherA2*/*ORCA* without any approximation of the crystal environment. Lighter coloured datasets have an *F*/σ(*F*) cut-off of 4 as applied in the reference.

We can compare our refinement of these two datasets with a recent publication, which used cluster charges to a distance of 8 Å and the B3PW91 functional for a refinement on the absolute structure factors instead of the intensities (Malaspina *et al.*, 2021*b* ▸). The reference refinement also used an *F*/σ(*F*) cut off of 4. Again, we did two separate refinements against the cut data and against the full data, which had been published before (Malaspina *et al.*, 2020 ▸). The resulting quality indicators can be found in Figs. 5 ▸ and 6 ▸. Obviously for these datasets the periodic refinement is significantly beneficial for both the agreement factor and the *X*—H distances. However, the reference hybrid functional description does produce smaller outliers for the *S*_12_ value.

Interestingly, the 8 Å cluster charge calculation obtained via *NoSpherA2*/*TONTO* also shows a better agreement in the bond distances compared with the reference, especially for the **HMa-Mg** dataset. At the same time, it does not quite reach the level of the periodic PAW-based refinement. A possible explanation could be that the refinement was against *F* ^2^ in *NoSpherA2* instead of *F* which was employed in the reference.

Both datasets show systematic features in the residual density around the atomic positions, which are similar for all refinements conducted within this work. This could be an indication that we are limited by an undescribed effect within the data. This would also fit to the fact that the overall differences are somewhat small. Finding and correcting for this effect should lead to larger differences overall. Nevertheless, PAW refinements for these datasets show a significant improvement, which would point to the benefit of periodic calculations for the description of charged species.

#### 3.3.3. Xylitol

For this dataset we will compare with a refinement which approximated the crystal environment by embedding the structure calculated by B3LYP into a 4 Å layer described by ELMOs, which was further surrounded by a layer treated by classical molecular mechanics (Wieduwilt *et al.*, 2021 ▸).

**Figure 7:**
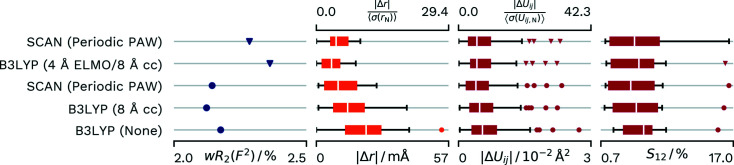
Differences in *wR*_2_(*F* ^2^) and agreement with neutron values for different density sources for HAR of **Xy**. Refinements marked with triangles do not include an extinction correction, while refinements marked with circles do. Distributions of parameters are displayed as box-whisker plots. SCAN (Periodic PAW) was conducted with our script and *GPAW*. The B3LYP (4 Å ELMO/8 Å cc) values are from the reference calculation from the literature (Wieduwilt *et al.*, 2021 ▸) and used ELMOs as a tool for quantum-mechanically calculating a 4 Å cluster in addition to 8 Å of cluster charges for the crystal approximation. B3LYP (8 Å cc) uses 8 Å of cluster charges and was refined with *NoSpherA2*/*TONTO*. B3LYP (None) was calculated with *NoSpherA2*/*ORCA* without any approximation of the crystal environment.

Refinement in the PAW-HAR scheme led to strong reflections with systematically underdetermined calculated intensities. There is some discrepancy in the literature on how to interpret this result: while the original publication (Madsen *et al.*, 2004 ▸) refined extinction to account for this fact, a more recent publication ascribed the result to crystal field effects and electron correlation and corrected for the underdetermination by X-ray restrained wavefunction fitting (Malaspina *et al.*, 2021*b* ▸). We agree with the original publication and refined an extinction correction as originally implemented in *SHELXL* (Sheldrick, 2015 ▸), while the reference calculation did not refine this additional parameter. Therefore, we can only determine a general trend in the relative performance of the two calculations. The difference in modelling might skew the results. A comparison between SCAN PAW-HAR calculations with or without extinction and the reference calculations without extinction and calculations in *NoSpherA2* can be found in Fig. 7 ▸.

In a direct comparison of the reference calculation and the PAW-HAR without extinction we can see a slightly lower *wR*_2_(*F* ^2^) and minimally improved agreement in the atomic displacement parameters for the periodic calculation, while there is a significant improvement in the *X*—H distance agreement for the reference calculation. Disagreement in distances increases with refinement of extinction while the agreement in displacements improves slightly. The crystallographic agreement factor profits greatly from extinction refinement. In contrast with other datasets, the cluster charge calculation shows an improved agreement factor compared with the periodic calculation. However, both the distance agreement and the agreement in atomic displacement parameters are lower. Unsurprisingly, the single-molecule calculation shows a higher *wR*_2_(*F* ^2^) and lower agreements in the H-atom distance and vibrational parameter.

Comparison without the consideration of extinction gives good reason to believe that the method reported in the reference would compare favourably with PAW-HAR for this dataset. However, we believe the ultimate answer can only be given if a refinement including extinction were published.

#### 3.3.4. Urea at 123 K

**Figure 8:**
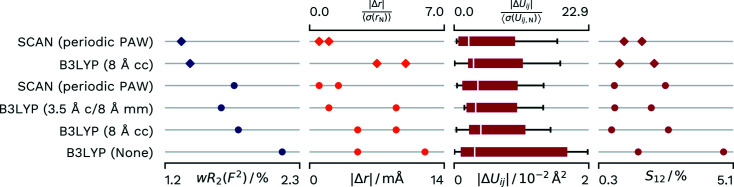
Differences in *wR*_2_(*F* ^2^) and agreement with neutron values for different density sources for HAR of **Urea**. The two independent values for *X*—H distances and H-atom *S*_12_ are displayed as points. Distributions of atomic displacement parameters are displayed as box-whisker plots. SCAN (Periodic PAW) was conducted with our script and *GPAW*. The B3LYP (3.5 Å c, 8 Å mm) values are from the reference calculation from the literature (Chodkiewicz *et al.*, 2020 ▸) and used a 3.5 Å cluster that was fully calculated and 8 Å of point multipoles for the crystal approximation. B3LYP (8 Å cc) uses 8 Å of cluster charges and was refined with *NoSpherA2*/*TONTO*. B3LYP (None) was calculated with *NoSpherA2*/*ORCA* without any approximation of the crystal environment. The refinements marked with diamonds were minimized with additional Gram–Charlier parameters.

As can be seen in Fig. 8 ▸, the refinement of urea does show a slight disadvantage in *wR*_2_(*F* ^2^) compared with the reference B3LYP (Chodkiewicz *et al.*, 2020 ▸) calculation which represents the crystal surroundings by a fully calculated 3.5 Å cluster of urea molecules and an 8 Å cluster of multipoles. Compared with this high-level computation, the PAW refinement also shows a minimally lower agreement in the derived anisotropic displacement parameters. However, the H-atom distances show higher agreement with deviations of only 0.001 and 0.003 Å, where the estimated standard deviation of the neutron bond lengths is 0.002 Å.

**Figure 9:**
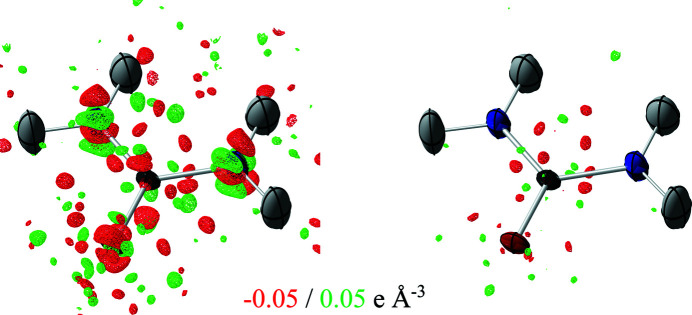
Difference electron-density plots for different refinements of urea. (Left) Refinement with SCAN PAW-HAR without Gram–Charlier expansion. (Right) Refinement with SCAN PAW-HAR with third (N, O) and fourth (N, O) degree Gram–Charlier expansion.

The final difference electron density is on a low level but not completely featureless for all refinements done in this paper (see left-hand image in Fig. 9 ▸). A probable reason for the features could be identified as anharmonic displacement. Accordingly, Gram–Charlier parameters of third and fourth order were refined for the N and O atoms, which were significant. The difference electron density significantly reduced in size around the atom positions. We also used *XDPDF* from the *XD2016* suite (Volkov *et al.*, 2016 ▸) to confirm that the refinement did not result in negative probability density for the atom positions. Further information and validation for the Gram–Charlier refinement can found in the supporting information (Section S6).

However, for the cluster-charge HAR in *NoSpherA2*/*TONTO* this improvement in the difference electron density was accompanied by a decrease in agreement with the neutron diffraction derived *X*—H distances and anisotropic displacement parameters.

In contrast, PAW-HAR with Gram–Charlier parameters resulted in an almost exact agreement of the calculated *X*—H distances with the neutron values, where the difference for each atom was about 0.001 Å. It also resulted in a slight improvement in the agreement of the anisotropic displacement parameters (see Fig. 8 ▸).

The urea structure demonstrates the performance of the PAW-HAR method in a best-case scenario. By improving the density description, we could resolve the anharmonic vibration and the *XHARPy* library enabled the refinement of the respective Gram–Charlier parameters.

### 3.4. Speed of the calculation

We can now compare the relative speed of PAW-HAR in its current implementation against calculations that used 8 Å of cluster charges in *NoSpherA2*/*TONTO*. In order to compare the two implementations directly, neither was pre-refined at a lower level. This meant, however, that with the exception of urea, the structures did not reach convergence according to the criteria of *OLEX2* with *NoSpherA2*/*TONTO* and 8 Å of cluster charges. For all of these refinements we reached a point where the difference in atomic parameters from the previous calculation, and the agreement factors, always remained at the same values. The first of these points was interpreted as convergence. For PAW-HAR, convergence according to the criteria of the *XHARPy* library was reached in all cases.

Of the five structures PAW-HAR was faster in three cases, while the cluster-charge calculation was faster in the remaining two (see Table 1 ▸) with the given settings.

Unsurprisingly, the most favourable comparison for PAW-HAR comes from the two hydrogen maleate structures. Both calculations included only the single Γ point in the atomic form factor calculation. Additionally, the large fragment in the cluster-charge calculation of the **HMa-Mg** structure provides the worst-case scenario for *TONTO*.

The other extreme is the urea structure with its very small fragment in the cluster-charge calculation and the very fine grid spacing and higher number of *k*-points in the PAW-HAR calculation. This is, however, also accompanied by a significant benefit for the description, both for the residual density and the determined distances (see Section 3.3.4).

In summary, the relative computation time is highly dependent on the selected settings and structures. This is remarkable as the PAW-HAR method takes the crystal environment into account quantum mechanically, while the point of comparison is the lower-level cluster-charge computation.

### 3.5. Comparison of aggregated parameters

We can now aggregate the agreement in *X*—H bond distances and anisotropic displacement parameters for all discussed structures in order to compare them. For this purpose, we defined hydrogen bonds along the narrow classical criterion with an *X*—H⋯*Y* bond, where *X* and *Y* are either N or O atoms. With this, the H atoms and their bonds can be divided into three separate groups. (i) The majority of H atoms are engaged in C—H bonds (21 atoms/bonds). Within the hydrogen bonds we distinguish (ii) those where the atoms do not share the same calculated fragment (inter *X*—H⋯*Y*, 16 atoms/bonds) and (iii) those where *X*—H donor and *Y* acceptor are within the same calculated fragment in the *NoSpherA2*-based calculations (intra *X*—H⋯*Y*, four atoms/bonds).

**Figure 10:**
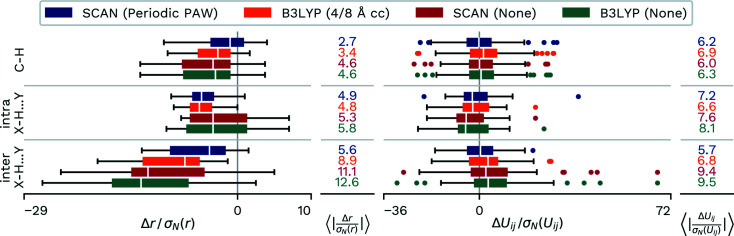
Differences in *X*—H bond distances and anisotropic displacement parameters divided by the estimated standard deviations from the neutron diffraction refinement. H atoms are grouped by bond type and position in the fragment in *NoSperA2*. Distributions are depicted as box-whisker plots. Additionally, absolute values over estimated standard deviations are averaged over all investigated H atoms. SCAN (Periodic PAW) was conducted with our script and *GPAW*. B3LYP (4/8 Å cc) uses 4/8 Å of cluster charges and was refined with *NoSpherA2*/*TONTO*. B3LYP/SCAN (None) were calculated with *NoSpherA2*/*ORCA* without any approximation of the crystal environment.

Additionally, we want to scale the deviations by the estimated standard deviations from the neutron refinement (σ_n_) values for both the distances and the atomic displacement parameters, in order to determine whether the differences are actually significant. Criteria aggregated in this way are depicted in Fig. 10 ▸.

For the C—H bonds, we can observe that there is an improvement in the obtained bond lengths if we take the crystal surroundings into consideration. The difference in improvement of the cluster-charge description versus PAW-HAR is smaller and lies below an estimated standard deviation at 0.7 σ_n_. The anisotropic displacement parameters do not profit from the cluster-charge description and the difference increases by 0.6 σ_n_ between the two calculations from the B3LYP functional. Additionally, we can observe that the deviations are no longer centred around zero. This is not the case for PAW-HAR and the scaled deviations in anisotropic displacement parameters are lower by 0.7 σ_n_ than the cluster-charge counterparts, which means the performance is close to that obtained with no crystal description. For the hydrogen bonds located within a fragment, we see identical performance for the bond lengths. Both methods are a significant improvement over the bond lengths determined without crystal surroundings. Only the cluster charge shows a significant improvement for anisotropic displacement parameters. As expected, the most significant differences between the methods can be observed for interfragmental hydrogen bonds. Not only is the improvement in the distance agreement from no crystal surroundings to cluster-charge description more than 2 σ_n_, PAW-HAR shows a further improvement of 3.4 σ_n_ over the cluster-charge result. The improvements are smaller but still significant for the anisotropic displacement parameters, with more than 2.6 σ_n_ improvement with the inclusion of cluster charges and a further 1.1 σ_n_ improvement from cluster charges to PAW-HAR.

Finally, the results also clearly show that the performance is not simply a result of the performance of the SCAN functional. The results show a clear distinction between the refinement using the periodic PAW density evaluation and the refinement not employing a description of the crystal surroundings for this functional.

## 4. Conclusion and outlook

We have successfully demonstrated the benefit of using periodic density functional theory calculations with the projector augmented wave method in Hirshfeld atom refinement. To this end we have developed a custom library in Python named *XHARPy*. An evaluation of a suite of functionals showed the best overall performance for the meta-generalized gradient approximation functionals SCAN and revSCAN for this method.

In the investigated structures we have shown a significant improvement over published calculations that neglected the crystal environment or treated it classically. A comparison against calculations where the crystal environment was emulated by cluster embedding showed neither a clear advantage nor disadvantage. Distances show a very high agreement, while the *wR*_2_(*F* ^2^) value is higher. Due to the different treatment of extinction, a final verdict on the second dataset is not possible at the moment, independent of the density description. In summary, we have established that PAW-HAR is a possible quantum-mechanical treatment of the crystal surroundings, at least on a par with other state-of-the-art approaches.

For structures where the periodic density functional theory calculation was limited to the Γ point, we could see significant speed-ups in comparison with the atomic form factor calculation in *NoSpherA2*/*TONTO*. As expected, the overall relative speed is highly dependent on the respective settings. On average, however, our method seems to be faster with larger structures. The inclusion of more *k*-points can lead to a longer duration of the Hirshfeld atom refinement, especially as the smaller molecules were also calculated at very fine real-space grids in PAW-HAR.

The overall success of our method demonstrates that the density calculation and partitioning on a rectangular grid instead of an atom-centred one can only have a small influence. The combination of expansion on the grid and fast Fourier transform is fast and reliable when the spherical frozen-core density is calculated separately. As a number of quantum chemistry programs for the solid state rely on rectangular grids, this opens up new sources for the density. In addition to the GPAW interface employed for this work, an experimental implementation for *QUANTUM ESPRESSO* is already available in the *XHARPy* library.

Additionally, periodic calculations with the projector augmented wave scheme are a viable tool for obtaining atomic form factors and deriving very accurate H-atom positions and accurate H-atom displacement parameters. From a practical point of view, the central benefit is the absence of a fragment dependency. There is no potential bias from fortunate or unfortunate selections of the calculated fragment and/or cluster radii. The calculated fragment is the complete unit cell. We have demonstrated that using only cluster charges leads to a worse performance compared with the PAW-HAR for H atoms located at the border of the calculated fragment.

Overall, we would state that the present approach offers great potential. This is the case both from a conceptual standpoint that a periodic system is calculated as such, and from the presented results. Now that the viability of the approach with the presented refinement library is established, the application to inherently periodic structures and highly charged species, especially when combined with other density partitioning methods, would be the logical next steps. The *XHARPy* library itself is flexible enough to accommodate such investigations.

The library can be downloaded from the repository at https://github.com/Niolon/XHARPy under the GPL-3.0 license.

## Supplementary Material

Crystal structure: contains datablock(s) A23_SHELXL_iam, A23_xHARPy_iam, A23_NoSpherA2_B3LYP_ORCA, A23_NoSpherA2_B3LYP_tonto, A23_NoSpherA2_PBE_ORCA, A23_NoSpherA2_SCAN_ORCA, A23_xharpy_SCAN, A23_xharpy_SCAN_iovs_cut, A23_xHARPy_BEEF-vdW, A23_xHARPy_BLYP, A23_xHARPy_PBE, A23_xHARPy_PW, A23_xHARPy_PW91, A23_xHARPy_RPBE, A23_xHARPy_TPSS, A23_xHARPy_revPBE, A23_xHARPy_revSCAN, A23_xHARPy_vdW-DF, A23_xHARPy_vdW-DF2, A23_xHARPy_RPBE_fd_rectangular, A23_xHARPy_RPBE_lcao_rectangular, A23_xHARPy_RPBE_lcao_spherical, HMa-8HQ_NoSpherA2_B3LYP_ORCA, HMa-8HQ_NoSpherA2_B3LYP_tonto, HMa-8HQ_NoSpherA2_PBE_ORCA, HMa-8HQ_NoSpherA2_SCAN_ORCA, HMa-8HQ_xharpy_SCAN, HMa-8HQ_xharpy_SCAN_iovscut, HMa-8HQ_xHARPy_BEEF-vdW, HMa-8HQ_xHARPy_BLYP, HMa-8HQ_xHARPy_PBE, HMa-8HQ_xHARPy_PW, HMa-8HQ_xHARPy_PW91, HMa-8HQ_xHARPy_RPBE, HMa-8HQ_xHARPy_TPSS, HMa-8HQ_xHARPy_revPBE, HMa-8HQ_xHARPy_revSCAN, HMa-8HQ_xHARPy_vdW-DF, HMa-8HQ_xHARPy_vdW-DF2, HMa-Mg_NoSpherA2_B3LYP_ORCA, HMa-Mg_NoSpherA2_B3LYP_tonto, HMa-Mg_NoSpherA2_PBE_ORCA, HMa-Mg_NoSpherA2_SCAN_ORCA, HMa-Mg_xharpy_SCAN, HMa-Mg_xharpy_SCAN_Iovs_cut, HMa-Mg_xHARPy_BEEF-vdW, HMa-Mg_xHARPy_BLYP, HMa-Mg_xHARPy_PBE, HMa-Mg_xHARPy_PW, HMa-Mg_xHARPy_PW91, HMa-Mg_xHARPy_RPBE, HMa-Mg_xHARPy_TPSS, HMa-Mg_xHARPy_revPBE, HMa-Mg_xHARPy_revSCAN, HMa-Mg_xHARPy_vdW-DF, HMa-Mg_xHARPy_vdW-DF2, Xy_NoSpherA2_B3LYP_ORCA, Xy_NoSpherA2_B3LYP_tonto, Xy_NoSpherA2_PBE_ORCA, Xy_NoSpherA2_SCAN_ORCA, Xy_xharpy_SCAN, Xy_xharpy_SCAN_noEXTI, Xy_xHARPy_BEEF-vdW, Xy_xHARPy_BLYP, Xy_xHARPy_PBE, Xy_xHARPy_PW, Xy_xHARPy_PW91, Xy_xHARPy_RPBE, Xy_xHARPy_TPSS, Xy_xHARPy_revPBE, Xy_xHARPy_revSCAN, Xy_xHARPy_vdW-DF, Xy_xHARPy_vdW-DF2, urea_NoSpherA2_B3LYP_ORCA, urea_NoSpherA2_B3LYP_tonto, urea_NoSpherA2_PBE_ORCA, urea_NoSpherA2_SCAN_ORCA, urea_NoSpherA2_SCAN_dijkl, urea_xharpy_SCAN, urea_xHARPy_SCAN_cijk, urea_xHARPy_SCAN_dijkl, urea_NoSpherA2_B3LYP_tonto_dijkl, urea_xHARPy_BEEF-vdW, urea_xHARPy_BLYP, urea_xHARPy_PBE, urea_xHARPy_PW, urea_xHARPy_PW91, urea_xHARPy_RPBE, urea_xHARPy_TPSS, urea_xHARPy_revPBE, urea_xHARPy_revSCAN, urea_xHARPy_vdW-DF, urea_xHARPy_vdW-DF2. DOI: 10.1107/S2052252522001385/fc5060sup1.cif

Structure factors: contains datablock(s) urea_NoSpherA2_SCAN_dijkl. DOI: 10.1107/S2052252522001385/fc5060urea_NoSpherA2_SCAN_dijklsup2.hkl

Structure factors: contains datablock(s) A23_NoSpherA2_SCAN_ORCA. DOI: 10.1107/S2052252522001385/fc5060A23_NoSpherA2_SCAN_ORCAsup3.hkl

Supporting Information on refinement quality. DOI: 10.1107/S2052252522001385/fc5060sup4.pdf

CCDC references: 2150631, 2150632, 2150633, 2150634, 2150635, 2150636, 2150637, 2150638, 2150639, 2150640, 2150641, 2150642, 2150643, 2150644, 2150645, 2150646, 2150647

## Figures and Tables

**Figure 6:**
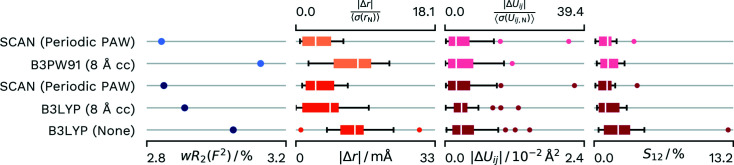
Differences in *wR*_2_(*F* ^2^) and agreement with neutron values for different density sources for HAR of **HMa-Mg**. Distributions of parameters are displayed as box-whisker plots. SCAN (Periodic PAW) was conducted with our script and *GPAW*. The B3PW91 (8 Å cc) values are from the reference calculation from the literature (Malaspina *et al.*, 2021*b* ▸) and used 8 Å of cluster charges for the crystal approximation. B3LYP (8 Å cc) uses 8 Å of cluster charges and was refined with *NoSpherA2*/*TONTO*. B3LYP (None) was calculated with *NoSpherA2*/*ORCA* without any approximation of the crystal environment. Lighter coloured datasets have an *F*/σ(*F*) cut-off of 4 as applied in the reference.

**Table 1:** Duration of calculations starting at the independent atom model Reference system: Dell Precision 3640, Intel Core i9-10900K CPU, 32 GB RAM. Used cores: 10. The faster calculation is marked in bold font for each dataset.

	**A23K**	**HMa-8HQ**	**HMa-Mg**	**Xy**	**Urea**
PAW-HAR	1:45 h	**2:01 h**	**0:54 h**	**3:20 h**	0:42 h
8 Å cc / 4 Å cc	**1:03 h**	8:23 h	13:17 h	3:38 h	**0:16 h**
