# Supplementary material for: Hirshfeld atom refinement based on projector augmented wave densities with periodic boundary conditions
Source: IUCrJ. 2022 Feb 26;9(Pt 2):286–97. doi: 10.1107/S2052252522001385 (PMC8895013; doi:10.1107/S2052252522001385)
Supplement: Supplementary file 4 [file m-09-00286-sup4.pdf]

IUCrJ

Volume 9 (2022)

Supporting information for article:

Hirshfeld atom refinement based on projector augmented wave densities with periodic boundary conditions

Paul Niklas Ruth, Regine Herbst-Irmer and Dietmar Stalke

Supporting information

S1. Discussion of the modified structure factor by Wall. 1
S1.1. The factor for Δ*x* and -Δ*x* do not offset each other for small Δ*x* 2
S1.2. Evaluation of LCAO derived densities Fourier Transformed on spherical and rectangular grids do not show a large difference. 2
S2. Validation of Refinement against SHELXL 3
S3. Parameters for the theoretical calculations 5
S3.1. PAW-HAR in GPAW 5
S3.2. Non-periodical refinement in NoSpherA2 6
S3.2.1. Tonto cluster charge calculations 6
S3.2.2. Orca isolated molecule calculations 7
S4. Jacob's ladder of Hirshfeld Atom Refinement 7
S5. Quality indicators for Hirshfeld Atom Refinement structures 8
S5.1. L-Alanine at 23 K 9
S5.2. 8-Hydroxyquinone Maleate at 15 K 13
S5.3. Hexaqua Magnesium Maleate at 15 K 20
S5.4. Xylitol at 122 K 27
S5.5. Urea at 123 K 33
S6. Gram-Charlier refinement of urea. 36
S6.1. Validation against Olex Refine 37
S6.2. Significance, positive probability density and Kuhs' rule 38
S6.3. Quality indicators for the Gram-Charlier Refinement 39
References 40

S1. Discussion of the modified structure factor by Wall.

In a previous application of PAW-DFT by Wall (Wall, 2016) the author postulated, that the multiplication by a phase factor "is not appropriate for translations by fractional grid points on a fixed rectilinear grid such as is used here." Instead, he proposed a different factor that can be found in the

IMPORTANT: this document contains embedded data - to preserve data integrity, please ensure where possible that the IUCr Word tools (available from http://journals.iucr.org/services/docxtemplate/) are installed when editing this document.

same paper in equation 3. His derivation was demonstrated with the one-dimensional case, culminating in the one-dimensional equation 15:

$$\frac{A_{\Delta x}(h)}{A(h)} = e^{\frac{-2\pi i\, h\, u_o}{N}}\left[(1-u_1) + e^{\frac{-2\pi i h}{N}}u_1\right] \tag{1}$$

In this equation $u = N\Delta x = u_o + u_1$, with u_0 being the integer component and $u_1$the fractional remainder in the range $0 \leq u_1 \leq 1$, h is our reciprocal index and N is the number of lattice points in our one-dimensional partition.

We however found the application of this work to lead to worse results in application. We want to give two additional reasons, why we finally opted to use a traditional phase factor with FFT in our study.

S1.1. The factor for Δ*x* and -Δ*x* do not offset each other for small Δ*x*

Let us now assume, we have only a small $\Delta x \leq 1/N$. In this case u_0 is zero and u_1 is $N\Delta x$ and the equation simplifies to:

$$\frac{A_{\Delta x}(h)}{A(h)} = (1-u_1) + e^{\frac{-2\pi i h}{N}}u_1 = 1 - N\Delta x + N\Delta x \cdot e^{\frac{-2\pi i h}{N}} \tag{2}$$

However, if we enter $-\Delta x$ into the equation with $|-\Delta x| \leq 1/N$, u_0 is -1 and $u_1 = 1 - N\Delta x$ and this then can be simplified to:

$$\frac{A_{-\Delta x}(h)}{A(h)} = 1 - N\Delta x + N\Delta x \cdot e^{\frac{2\pi i h}{N}} \tag{3}$$

The product $A_{\Delta x}(h) \cdot A_{-\Delta x}(h)$ should be one so that the operation is reversible, but this is not the case. For N=10, Δx = 0.05 and h = 1 the product becomes:

$$\frac{A_{0.05}(1)}{A(1)} \cdot \frac{A_{-0.05}(1)}{A(1)} = (-1)^{\frac{2}{5}} \cdot (4.25 - 4.75(-1)^{\frac{2}{5}} - 0.5(-1)^{\frac{1}{5}}$$

To us, the reversibility issue might cause issues for convergence. Note however that this in an issue with Walls procedure, not a rigorous theoretical derivation.

Additionally, in contrast to the pilot study of Wall, we applied the Hirshfeld procedure iteratively Even if there is a slight deviation, there should be no difference at convergence, *i.e.* $\Delta x = 0$.

S1.2. Evaluation of LCAO derived densities Fourier Transformed on spherical and rectangular grids do not show a large difference.

GPAW does offer the possibility of using different descriptions for the electron density. The description as a linear combination of atomic orbitals does offer the possibility to compare the expansion of the atomic densities on the rectangular grid with an expansion onto a spherical grid. We can therefore directly compare the difference in results from the two expansions.

The expansion was written in Python using the grid implementation of HORTON (Verstraelen *et al.*, 2015) with the integration of HORTON used for discrete Fourier Transform on the spherical grid.

The dataset we used for comparison was the L-Alanine at 23K. As the LCAO calculation in GPAW is only available for calculations on the GGA level, the RPBE functional was used for comparison. Grid-spacing for wavefunction calculations was 0.12 Å. Expansion on the rectangular grid was done with four-fold interpolation resulting in a grid of 0.03 Å. Atomic grids of the "insane" preset of HORTON were used for spherical expansion.

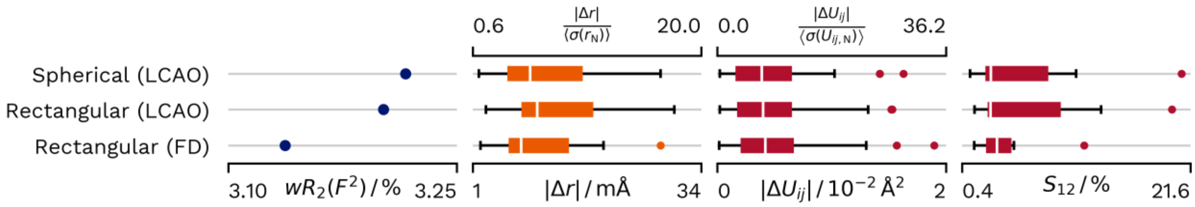

Figure S1 Differences in $wR_2(F^2)$ and agreement to neutron values for different expansions on grids for the calculation of atomic form factors. Distribution of parameters are displayed as box-whisker plots. All calculations were done using the RPBE functional.

The resulting quality indicators of the different refinement can be found in Figure S1. If we compare the two LCAO refinements, the $wR_2(F^2)$ is barely lower in the rectangular grid Fourier transform, the agreement to neutron data increases for the spherical grid Fourier transform.

However, using a finite difference real space description of the density shows a significantly larger benefit. This means from a practical point of view, even if there was a more beneficial alternative to the phase, its effect should be small. If we would use densities described as a linear combination of atomic orbitals with a spherical grid Fourier transform, we would discard the more beneficial density description, as well as the possibility to calculate densities with meta-GGA functionals.

As a consequence of all the afore mentioned points, this work uses a rectangular grid description as well as the traditional phase expression.

S2. Validation of Refinement against SHELXL

We used a completely new implementation within PYTHON for the refinement. In order to be sure that the implementation is actually correct, we compared IAM calculations of the 23 K L-Alanine dataset with the Python script to IAM calculations in SHELXL (Sheldrick, 2015). The resulting atomic parameters can be found in Tables S2 and S3. For all atoms the resulting parameters and estimated standard deviations are identical.

Table S1 Comparison of the fractional position parameters as well as the U_{iso} or U_{equiv} in Å^2 for the structure **A23K** calculated by the independent model from SHELXL as well as our script.

Atom	Program	*x*	*y*	*z*	U_{iso} or U_{equiv}
O1	SHELXL	0.72707(7)	0.08373(3)	0.62435(8)	0.00693(7)
	Ours	0.72707(7)	0.08373(3)	0.62435(8)	0.00693(7)
O2	SHELXL	0.44089(7)	0.18410(3)	0.76127(8)	0.00670(6)
	Ours	0.44089(7)	0.18410(3)	0.76127(8)	0.00670(6)
N1	SHELXL	0.64730(8)	0.13742(4)	0.18282(9)	0.00565(7)
	Ours	0.64730(8)	0.13742(4)	0.18282(9)	0.00565(7)
H1	SHELXL	0.6980(19)	0.0649(9)	0.189(2)	0.031(3)
	Ours	0.6980(19)	0.0649(9)	0.189(2)	0.031(3)
H2	SHELXL	0.7687(17)	0.1828(8)	0.201(2)	0.017(3)
	Ours	0.7687(17)	0.1828(8)	0.201(2)	0.017(3)
H3	SHELXL	0.579(2)	0.1459(9)	0.016(2)	0.030(3)
	Ours	0.579(2)	0.1459(9)	0.016(2)	0.030(3)
C1	SHELXL	0.46619(9)	0.16105(4)	0.35487(10)	0.00498(7)
	Ours	0.46619(9)	0.16105(4)	0.35487(10)	0.00498(7)
H4	SHELXL	0.4234(15)	0.2399(7)	0.3434(19)	0.007(2)
	Ours	0.4234(15)	0.2399(7)	0.3434(19)	0.007(2)
C2	SHELXL	0.25989(10)	0.09073(5)	0.30330(11)	0.00745(8)
	Ours	0.25989(10)	0.09073(5)	0.30330(11)	0.00745(8)
H5	SHELXL	0.2018(18)	0.1088(8)	0.152(2)	0.016(3)
	Ours	0.2018(18)	0.1088(8)	0.152(2)	0.016(3)
H6	SHELXL	0.1439(17)	0.1076(8)	0.423(2)	0.013(3)
	Ours	0.1439(17)	0.1076(8)	0.423(2)	0.013(3)
H7	SHELXL	0.3016(16)	0.0097(8)	0.309(2)	0.018(3)
	Ours	0.3016(16)	0.0097(8)	0.309(2)	0.018(3)
C3	SHELXL	0.55399(9)	0.14080(4)	0.59994(9)	0.00469(7)
	Ours	0.55399(9)	0.14080(4)	0.59994(9)	0.00469(7)

Table S2 Comparison of the anisotropic atomic displacement parameters of **A23K** in Å^2 calculated by the independent model from SHELXL as well as our script.

Atom	Program	U_{11}	U_{22}	U_{33}	U_{23}	U_{13}	U_{12}
O1	SHELXL	0.00678(13)	0.00706(13)	0.00696(16)	0.00064(12)	-0.00055(12)	0.00218(11)
	Ours	0.00678(13)	0.00706(13)	0.00696(16)	0.00064(12)	-0.00055(12)	0.00218(11)
O2	SHELXL	0.00797(13)	0.00792(13)	0.00421(15)	-0.00072(11)	0.00098(12)	0.00162(12)
	Ours	0.00797(13)	0.00792(13)	0.00421(15)	-0.00072(11)	0.00098(12)	0.00162(12)
N1	SHELXL	0.00581(14)	0.00651(14)	0.00463(16)	-0.00022(12)	0.00065(13)	0.00014(12)
	Ours	0.00581(14)	0.00651(14)	0.00463(16)	-0.00022(12)	0.00065(13)	0.00014(12)
C1	SHELXL	0.00556(16)	0.00544(14)	0.00394(18)	-0.00004(12)	0.00001(14)	0.00050(13)
	Ours	0.00556(16)	0.00544(14)	0.00394(18)	-0.00004(12)	0.00001(14)	0.00050(13)
C2	SHELXL	0.00630(17)	0.00946(17)	0.0066(2)	-0.00047(15)	-0.00060(15)	-0.00125(15)
	Ours	0.00630(17)	0.00946(17)	0.0066(2)	-0.00047(15)	-0.00060(15)	-0.00125(15)
C3	SHELXL	0.00562(15)	0.00428(13)	0.00419(17)	0.00007(12)	-0.00041(14)	-0.00044(13)
	Ours	0.00562(15)	0.00428(13)	0.00419(17)	0.00007(12)	-0.00041(14)	-0.00044(13)

S3. Parameters for the theoretical calculations

S3.1. PAW-HAR in GPAW

If not noted differently all calculations used the finite difference (FD) mode in GPAW. The convergence criterion for the density was tightened to 10^{-7}. If applied, Monkhorst-Pack grids (Monkhorst & Pack, 1976) were shifted to include the Γ point. If the Monkhorst-Pack grid is listed as Γ, the calculation only included a reciprocal space calculation at the origin of the reciprocal grid. In deviation to the default, full symmetry was considered. The Hirshfeld Atom Refinement cycle was employed until the difference of positions was under 10^{-6} Å. The Hirshfeld partitioning and atomic form factor calculation was calculated on a twice interpolated grid from the given wavefunction grid. This means, that the grid spacing for the FFT was one fourth of the given value. Additional parameters for the individual calculations are listed in Table S3.

Table S3 Parameters for the calculation of the individual datasets.

Dataset	Grid-spacing	Monkhorst-Pack grid
**A23K**	0.150 Å	(2,2,2)
**HMa-8HQ**	0.150 Å	Γ
**HMa-Mg**	0.150 Å	Γ
**Xy**	0.175 Å	(3,3,3)
**Urea**	0.100 Å	(3,3,3)

S3.2. Non-periodical refinement in NoSpherA2

For **A23K**, **HMa-8HQ** and **Xy** the fragment was the asymmetric unit itself. For **Urea**, the molecule was completed by applying the mirror plane that lies in the C,O axis but not in the complete molecule. For the **HMa-Mg** dataset the inversion centre on the Magnesium ion was applied to complete the hexaqua magnesium unit. In order to keep the overall inversion symmetry, the operation was applied to the maleate ion as well.

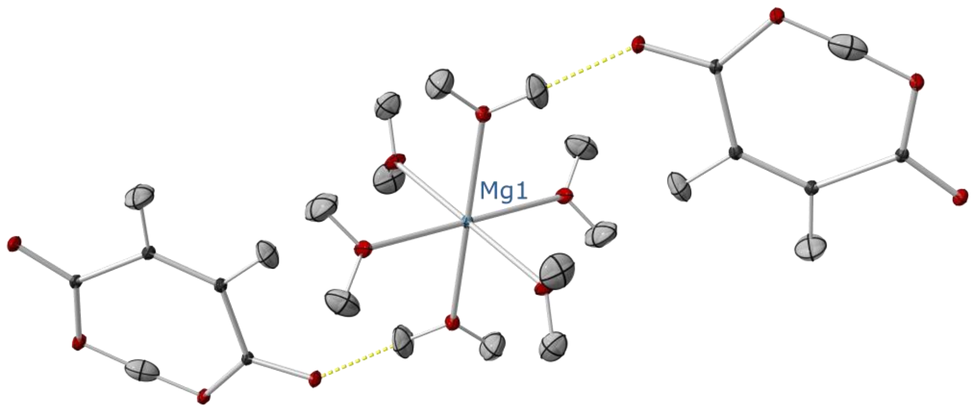

Figure S2 Depiction of the employed hexaqua magnesium maleate fragment, at the example of the Tonto calculation. The marked Mg1 atom is located on the inversion centre that was applied to the atoms of the asymmetric unit.

S3.2.1. Tonto cluster charge calculations

All structures were refined with the B3LYP functional and the def2-TZVPP basis set. Integration accuracy was set to high. Clusters were always completed. Structures were pre-refined using 4 Å of

cluster charges and a DIIS convergence criterion of 10^{-2}. The final refinement for all structures except **A23K** for was done with 8 Å of cluster charges and completed clusters. The DIIS convergence criterion was 10^{-4}. Due to convergence problems **A23K** used 10^{-5} as the convergence criterion, however full convergence could not be reached within 20 cycles. Additionally, as the overall performance of 4 Å of cluster charges was superior to the larger cluster, the smaller radius was used for the final comparison to other **A23K** refinements.

S3.2.2. Orca isolated molecule calculations

Structures were refined with the noted functional and the def2-TZVPP basis set. Integration accuracy was set to high. The SCF Threshold was set to "VeryTightSCF" the SCF Strategy was set to NormalConv.

S4. Jacob's ladder of Hirshfeld Atom Refinement

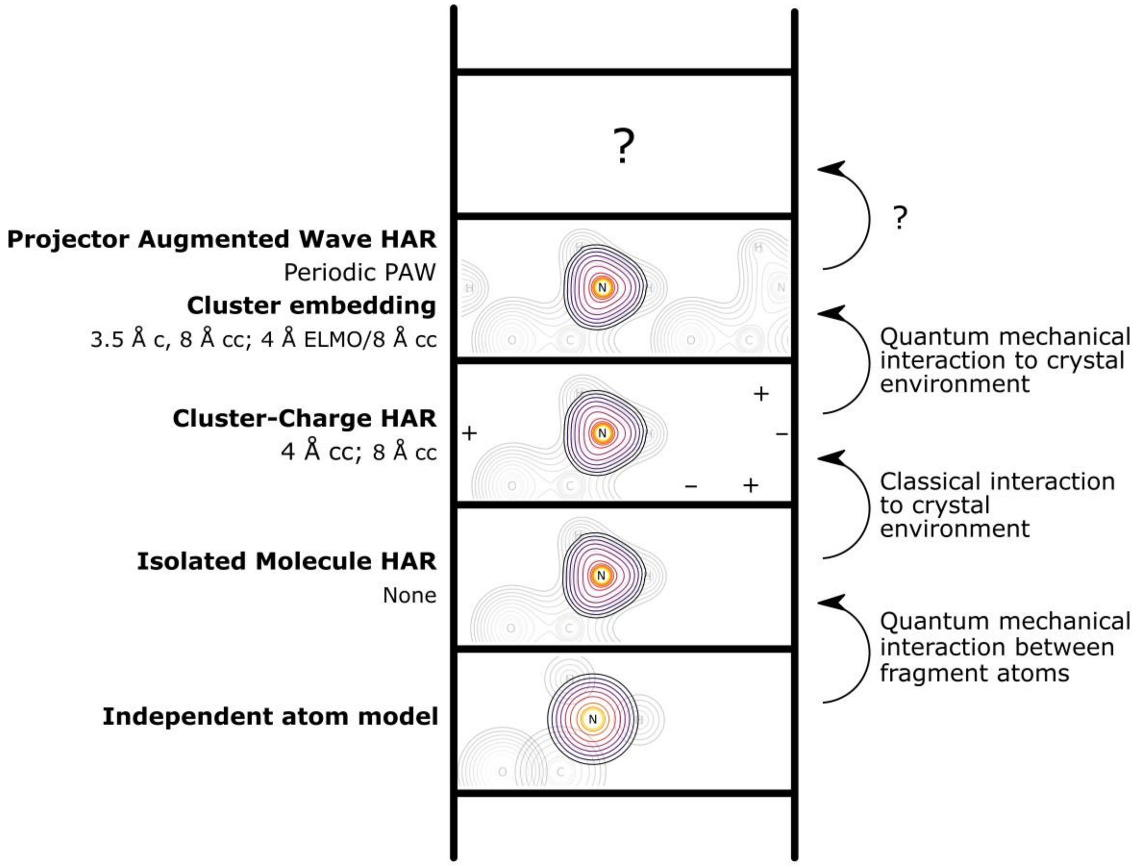

Figure S3 Jacob's ladder of Hirshfeld Atom Refinement, with increasing accuracy from bottom to top. Non-bold face naming on the left side is corresponding to the naming in Figures 4 – 7 in the publication. Reproduced after Wieduwilt *et al.* (2021).

S5. Quality indicators for Hirshfeld Atom Refinement structures

In addition to the quality indicators listed in the paper, the following indicators were evaluated for the evaluation of the agreement of the hydrogen atom description:

wRMSD(Δ*r*): This quality indicator is the weighted root mean square difference between the *X*–H bond distances derived from neutron diffraction and X-ray diffraction. The weighting employed is the combined estimated standard deviations of the X-ray and neutron derived values leading to the formula:

$$\mathrm{wRMSD}(\Delta r) = \left\langle \frac{(r_{\mathrm{X}} - r_{\mathrm{n}})^2}{\sigma^2(r_{\mathrm{X}}) + \sigma^2(r_{\mathrm{n}})} \right\rangle^{1/2} \tag{4}$$

wRMSD(Δ*U*$_{ij}$): This quality indicator is the weighted root mean square difference between the atomic displacement parameters derived from neutron diffraction and X-ray diffraction. The weighting employed is combined estimated standard deviations of the X-ray and neutron derived values leading to the formula:

$$\mathrm{wRMSD}(\Delta \mathrm{U_{ij}}) = \left\langle \frac{\left(U_{\mathrm{X}}^{ij} - U_{\mathrm{n,c}}^{ij}\right)^2}{\sigma^2\left(U_{ij,X}\right) + \sigma^2\left(U_{\mathrm{n,c}}^{ij}\right)} \right\rangle^{1/2} \tag{5}$$

‹ΔV/V$_{\mathbf{n}}$›: To determine whether the deviations between the anisotropic descriptions are random or systematic, we determined the difference in between calculated volumes of the probability function from neutron and Hirshfeld Atom Refinement divided by the respective volume of the neutron probability function. If $\mathbf{U}_{\mathrm{X}}$ and $\mathbf{U}_{\mathrm{n}}$ are the Cartesian displacement matrices from X-ray and neutron refinement respectively, the relative difference in volume can be calculated as:

$$\langle \Delta \mathrm{V}/V_{\mathrm{N}} \rangle = \langle (\det \mathbf{U}_{\mathrm{X}} - \det \mathbf{U}_{\mathrm{n}}) / \det \mathbf{U}_{\mathrm{n}} \rangle \tag{6}$$

Additionally aggregated DRK(Adam Stash, 2007) and Henn-Meindl-plots / residual density plots (Meindl & Henn, 2008) have been produced for all structures and functionals investigated.

S5.1. L-Alanine at 23 K

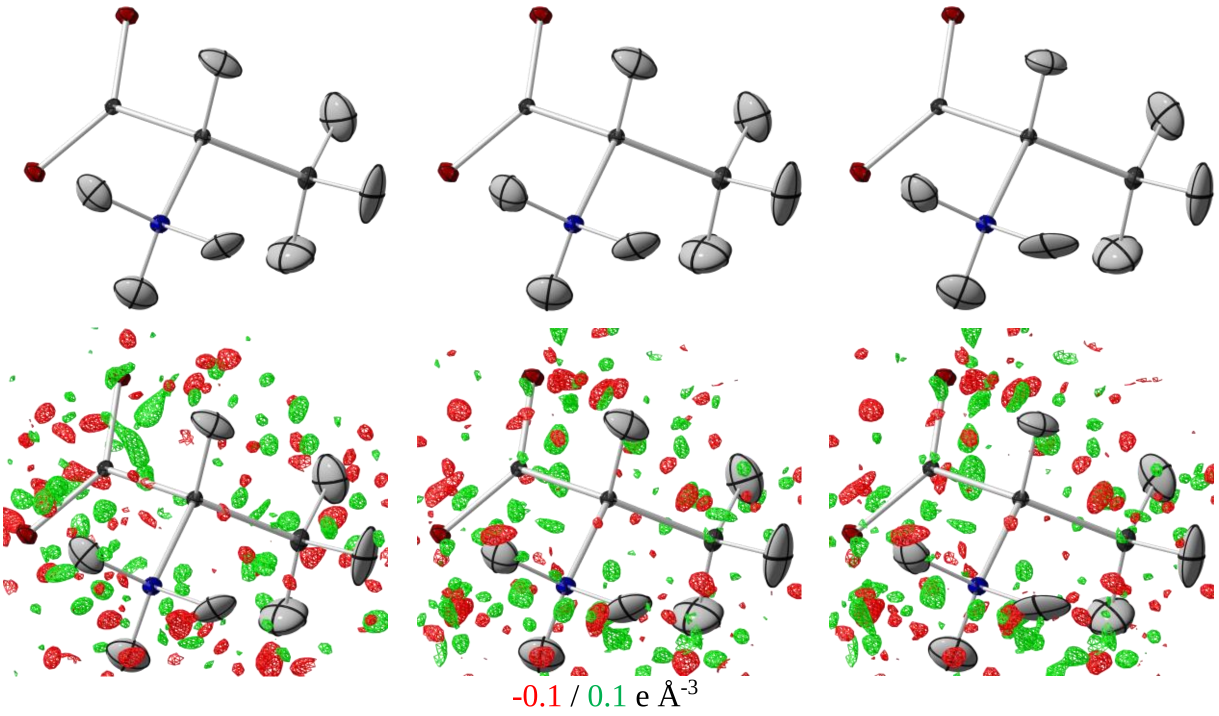

Figure S4 Structure images for **A23K**: Left: Resulting structure from the PAW-HAR refinement with the SCAN functional. Centre: Resulting structure of the refinement with B3LYP and 4 Å of cluster charges in tonto. Right: Resulting structure of the refinement of the isolated molecule density calculation in Orca using the B3LYP functional. All anisotropic displacement parameters are depicted at the 50 % probability level. For each structure there is an image without (top) and with (bottom) difference electron density.

Table S4 Aggregated quality indicators the agreement in X-ray intensities and hydrogen atom description from for all density sources for the PAW-HAR calculations of the L-Alanine at 23 K (**A23K**) dataset

functional	$wR_2(F^2)$	*GOF*	Δr	$\lvert\Delta r\rvert$	*wRMSD*	ΔU_{ij}	$\lvert\Delta U_{ij}\rvert$	*wRMSD*	S_{12}	V_X/V_N
	%		mÅ	mÅ	(Δr)	10^{-2} Å^2	10^{-2} Å^2	(ΔU_{ij})	%	
PW	3.31	1.18	-8	11	2.22	0.0	0.5	1.56	NPD	0.4
±			14	11		0.6	0.3		NPD	0.3
BLYP	3.20	1.14	-12	13	2.36	0.3	0.5	1.54	4.6	1.3
±			12	11		0.6	0.4		2.6	0.5
PW91	3.17	1.13	-9	11	2.20	0.2	0.5	1.47	5.1	1.0
±			11	10		0.5	0.3		2.6	0.4
PBE	3.16	1.12	-9	11	2.37	0.2	0.5	1.45	4.7	1.1

±			11	10		0.5	0.3		2.1	0.4
revPBE	3.12	1.11	-10	11	2.33	0.3	0.5	1.50	3.8	1.3
±			11	10		0.5	0.4		1.3	0.4
RPBE	3.11	1.11	-10	11	2.33	0.3	0.5	1.52	4.1	1.4
±			11	9		0.5	0.4		1.5	0.5
TPSS	3.14	1.12	-11	11	2.26	0.3	0.5	1.49	3.7	1.5
±			11	10		0.5	0.4		1.3	0.5
SCAN	3.06	1.09	-7	9	1.84	0.2	0.4	1.42	3.9	1.1
±			9	8		0.5	0.3		1.7	0.4
revSCAN	3.03	1.08	-5	8	1.56	0.2	0.4	1.39	3.5	1.2
±			8	6		0.5	0.3		1.5	0.4
vdW-DF	3.14	1.12	-14	14	2.63	0.4	0.5	1.65	3.7	1.8
±			11	10		0.6	0.4		1.2	0.5
vdW-DF2	3.22	1.15	-16	16	2.84	0.4	0.6	1.77	4.5	1.8
±			12	12		0.6	0.5		2.1	0.6
BEEF-vdW	3.10	1.11	-11	12	2.38	0.4	0.5	1.70	3.6	1.8
±			10	9		0.6	0.4		1.1	0.5

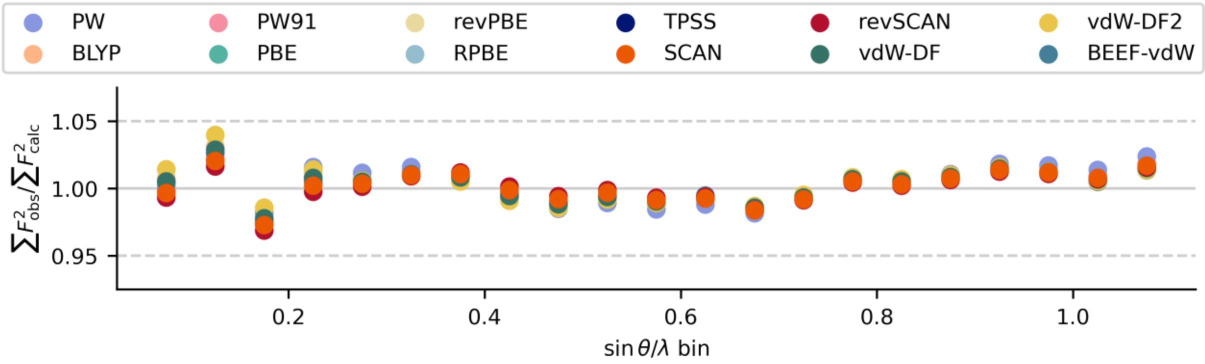

Figure S5 DRK plots of all periodic PAW calculations in this work for the L-Alanine at 23 K (**A23K**) dataset.

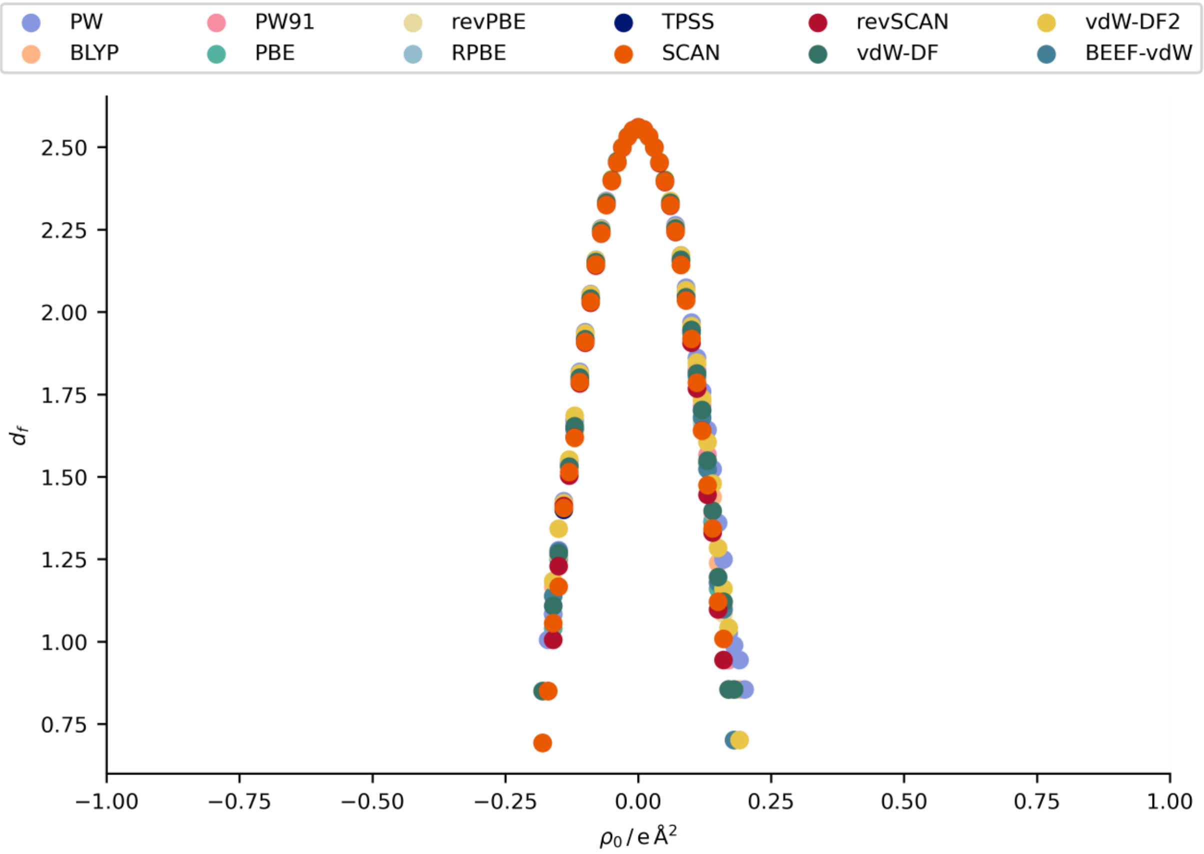

Figure S6 Henn-Meindl plots of all periodic PAW calculations in this work for the L-Alanine at 23 K (**A23K**) dataset.

Table S5 Aggregated quality indicators the agreement in X-ray intensities and hydrogen atom description from for all density sources for the non-periodical calculations of the L-Alanine at 23 K (**A23K**) dataset

functional	$wR_2(F^2)$ %	*GOF*	Δr mÅ	$\|\Delta r\|$ mÅ	*wRMSD* (Δr)	ΔU_{ij} 10^{-2} Å^2	$\|\Delta U_{ij}\|$ 10^{-2} Å^2	*wRMSD* (ΔU_{ij})	S_{12} %	V_X/V_N
			HAR non-periodic with 4 Å of cluster charges in Tonto							
B3LYP	3.06	1.09	-11.7	12	2.32	0.3	0.5	1.73	3.8	1.7
±			8.6	9		0.6	0.4		1.9	0.5
			HAR non-periodic isolated molecule conditions in ORCA							
B3LYP	3.17	1.13	-10	12	2.44	0.3	0.6	1.94	7.7	1.3
±			11	9		0.7	0.6		10.1	0.5
PBE	3.24	1.15	-10	12	2.52	0.3	0.6	2.03	7.7	1.1
±			12	10		0.8	0.6		7.7	0.4
SCAN	3.12	1.11	-8	10	2.00	0.3	0.6	1.95	7.3	1.3
±			10	7		0.7	0.6		9.0	0.4

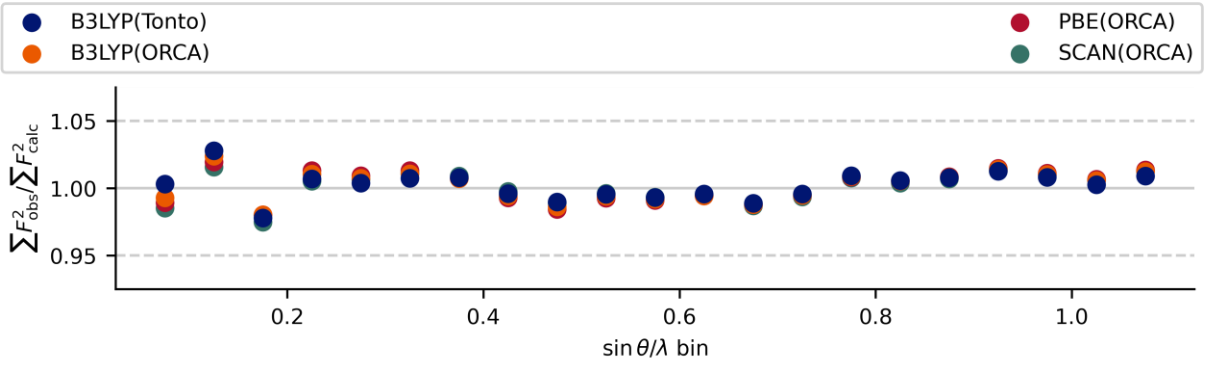

Figure S7 DRK plots of all non-periodic calculations in this work for the L-Alanine at 23 K (**A23K**) dataset.

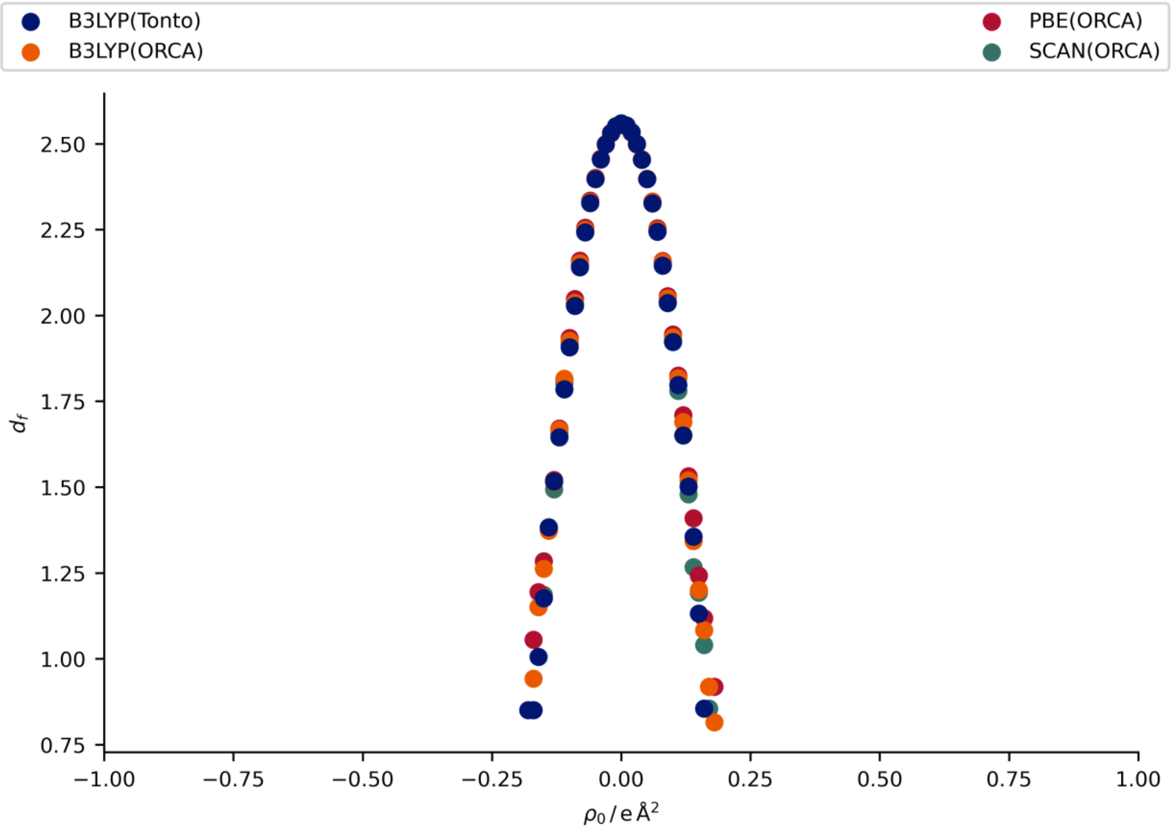

Figure S8 Henn-Meindl plots of all non-periodic calculations in this work for the L-Alanine at 23 K (**A23K**) dataset.

S5.2. 8-Hydroxyquinone Maleate at 15 K

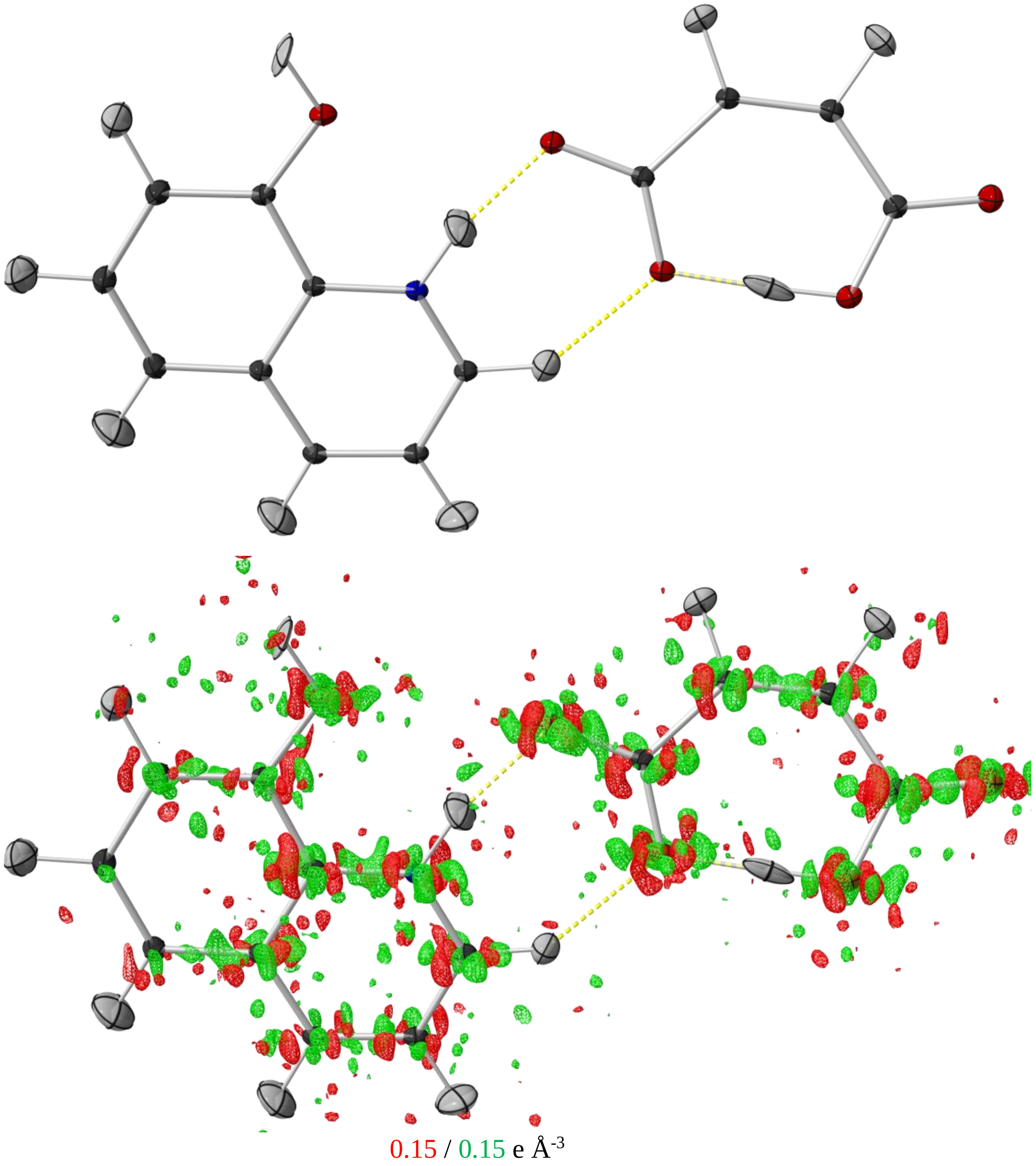

Figure S9 Resulting structure for **HMa-8HQ** from the PAW-HAR refinement with the SCAN functional without (top) and with (bottom) difference electron density. All anisotropic displacement parameters are depicted at the 50 % probability level.

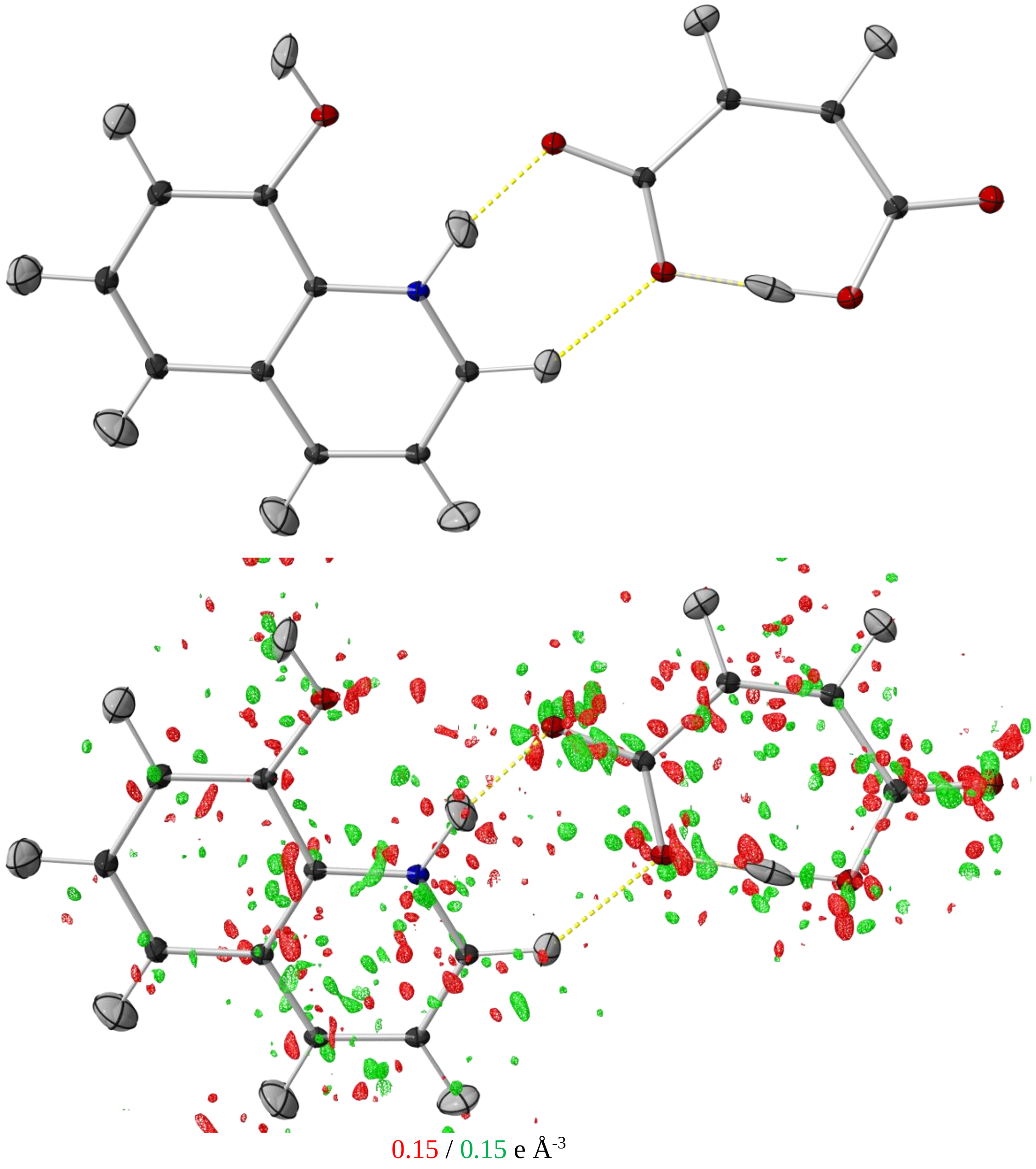

Figure S10 Resulting structure for **HMa-8HQ** from the refinement with 8 Å of cluster charges in tonto using the B3LYP functional without (top) and with (bottom) difference electron density. All anisotropic displacement parameters are depicted at the 50 % probability level.

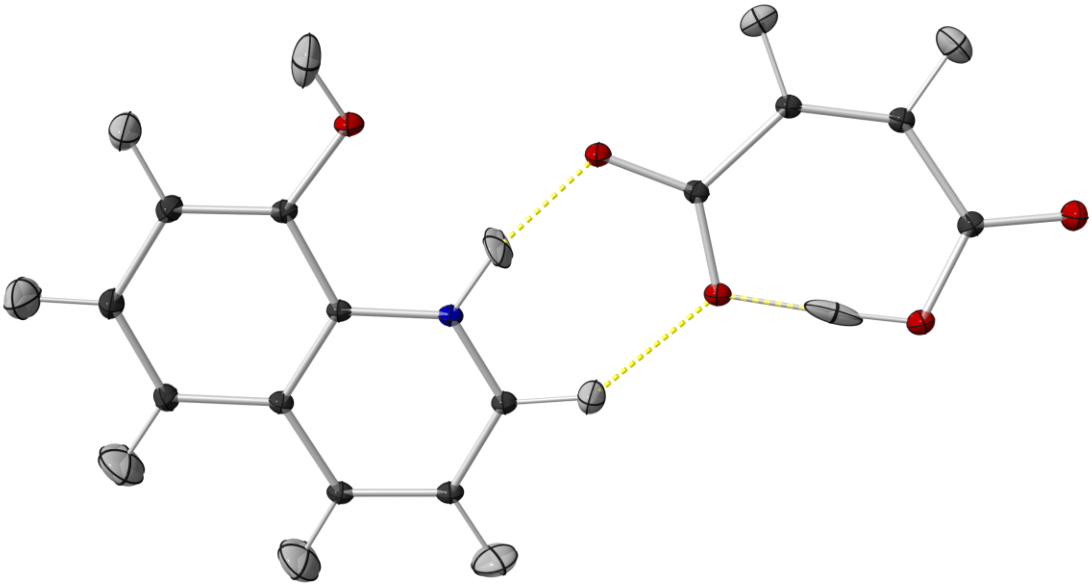

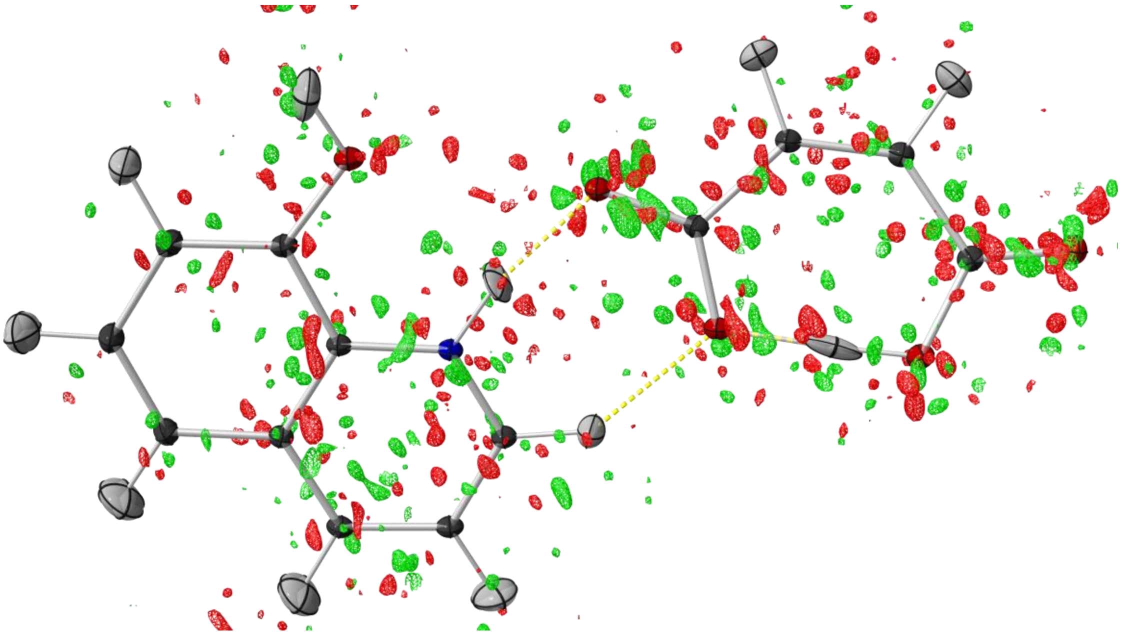

0.15 / 0.15 e Å^{-3}

Figure S11 Resulting structure for **HMa-8HQ** from the refinement of the isolated molecule density calculation in Orca using the B3LYP functional without (top) and with (bottom) difference electron density. All anisotropic displacement parameters are depicted at the 50 % probability level.

Table S6 Aggregated quality indicators the agreement in X-ray intensities and hydrogen atom description from for all density sources for the PAW-HAR calculations of the 8-hydroxyquinone hydrogen maleate at 15 K (**HMa-8HQ**) dataset

functional	$wR_2(F^2)$ %	*GOF*	Δr mÅ	$\|\Delta r\|$ mÅ	*wRMSD* (Δr)	ΔU_{ij} 10^{-2} Å^2	$\|\Delta U_{ij}\|$ 10^{-2} Å^2	*wRMSD* (ΔU_{ij})	S_{12} %	V_X/V_N
PW	4.11	1.52	-13	15	2.78	-0.3	0.7	2.50	NPD	0.3
±			17	15		0.9	0.6		NPD	0.2
BLYP	4.08	1.50	-14	15	2.69	-0.0	0.5	2.00	4.7	0.7
±			15	14		0.7	0.5		6.5	0.3
PW91	4.07	1.50	-12	13	2.62	-0.1	0.5	2.04	5.9	0.6
±			14	13		0.7	0.5		7.8	0.3
PBE	4.07	1.50	-12	13	2.58	-0.1	0.5	2.04	5.9	0.6
±			14	13		0.7	0.5		7.8	0.3
revPBE	4.06	1.50	-11	12	2.42	-0.0	0.5	1.92	5.1	0.7
±			13	12		0.7	0.5		7.4	0.3
RPBE	4.06	1.50	-11	12	2.36	-0.0	0.5	1.92	5.0	0.8
±			13	12		0.7	0.5		7.3	0.3
TPSS	4.05	1.49	-11	12	2.36	-0.0	0.5	1.85	4.5	0.8
±			13	12		0.7	0.5		6.3	0.3
SCAN	4.03	1.49	-6	9	1.73	-0.1	0.5	1.81	4.9	0.7
±			11	9		0.6	0.4		7.1	0.3
revSCAN	4.04	1.49	-5	7	1.42	-0.1	0.5	1.84	5.7	0.7
±			9	7		0.6	0.4		8.8	0.3
vdW-DF	4.07	1.50	-14	14	2.47	0.1	0.5	1.93	3.9	0.9
±			13	12		0.7	0.5		5.3	0.3
vdW-DF2	4.12	1.52	-15	16	2.85	0.1	0.6	1.94	3.9	0.9
±			15	15		0.8	0.5		5.0	0.3
BEEF-vdW	4.07	1.50	-11	12	2.24	0.1	0.5	1.82	3.9	0.9
±			12	11		0.7	0.4		5.6	0.3

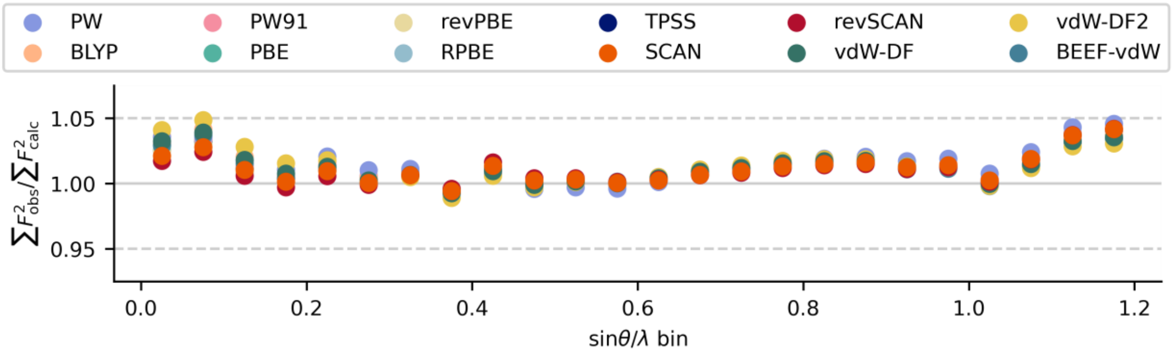

Figure S12 DRK plots of all periodic PAW calculations in this work for the 8-hydroxyquinone hydrogen maleate at 15 K (**HMa-8HQ**) dataset.

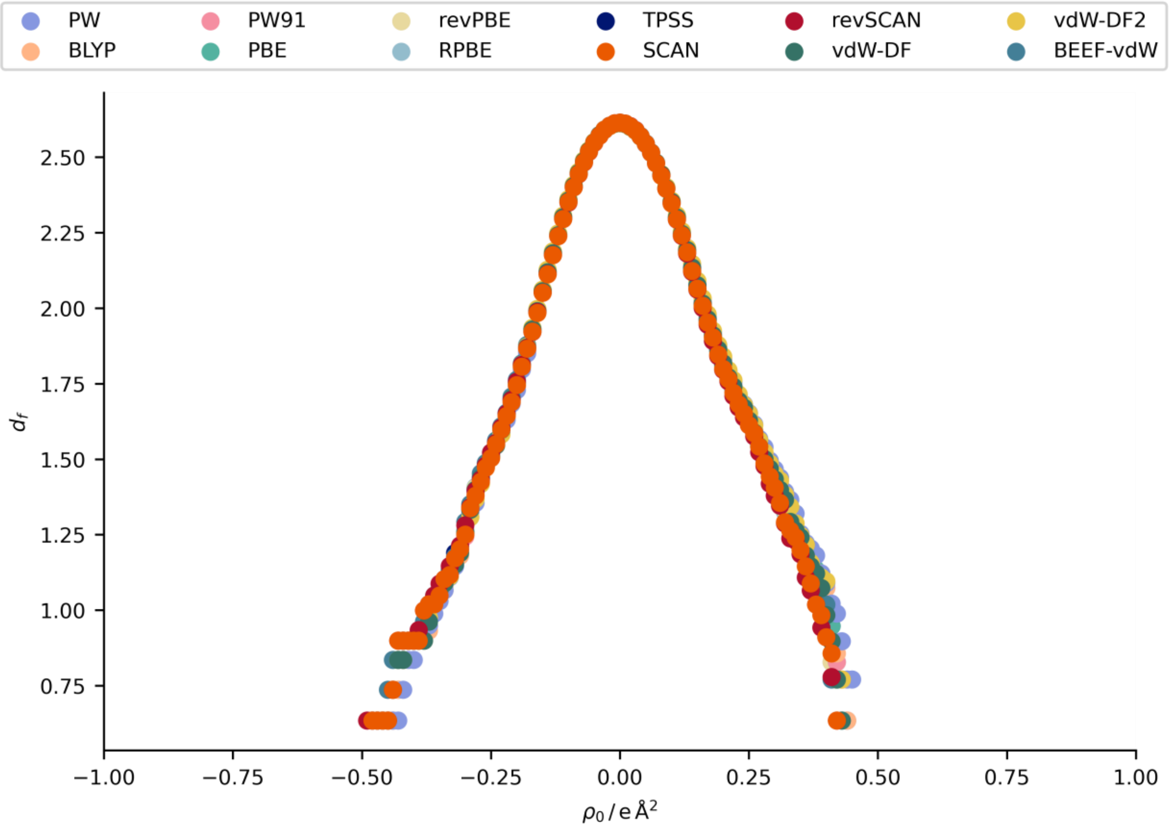

Figure S13 Henn-Meindl plots of all periodic PAW calculations in this work for the 8-hydroxyquinone hydrogen maleate at 15 K (**HMa-8HQ**) dataset.

Table S7 Aggregated quality indicators the agreement in X-ray intensities and hydrogen atom description from for all density sources for the non-periodical calculations of the 8-hydroxyquinone hydrogen maleate at 15 K (**HMa-8HQ**) dataset

functional	$wR_2(F^2)$ %	*GOF*	Δr mÅ	$\lvert\Delta r\rvert$ mÅ	*wRMSD* (Δr)	ΔU_{ij} 10^{-2} Å^2	$\lvert\Delta U_{ij}\rvert$ 10^{-2} Å^2	*wRMSD* (ΔU_{ij})	S_{12} %	V_X/V_N
HAR non-periodic with 8 Å of cluster charges in Tonto										
B3LYP	4.07	1.50	-11.2	13	2.33	-0.0	0.5	1.72	3.0	0.9
±			11.4	10		0.6	0.4		2.9	0.3
HAR non-periodic isolated molecule conditions in ORCA										
B3LYP	4.06	1.50	-14	15	2.89	-0.1	0.5	1.92	4.2	0.8
±			14	13		0.7	0.5		4.4	0.3
PBE	4.09	1.51	-17	17	3.34	-0.1	0.6	2.13	5.3	0.6
±			15	15		0.8	0.5		5.7	0.3
SCAN	4.05	1.49	-13	13	2.54	-0.1	0.5	1.92	4.4	0.7
±			12	11		0.7	0.5		4.5	0.3

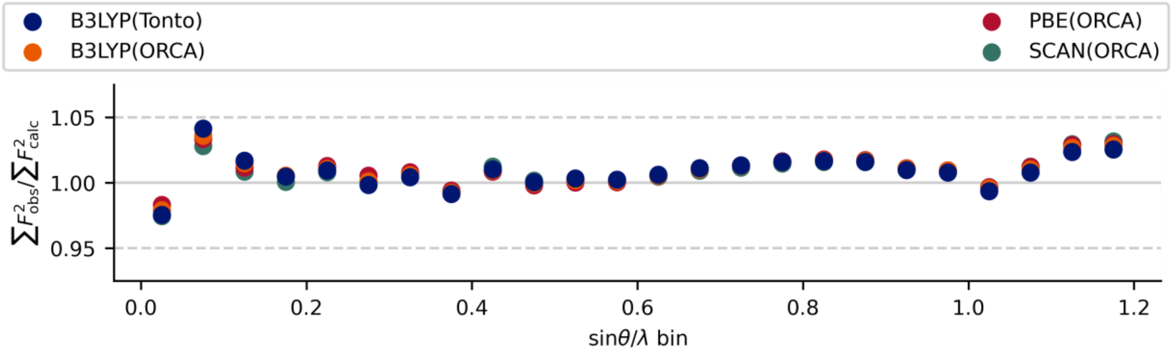

Figure S14 DRK plots of all non-periodic calculations in this work for the 8-hydroxyquinone hydrogen maleate at 15 K (**HMa-8HQ**) dataset.

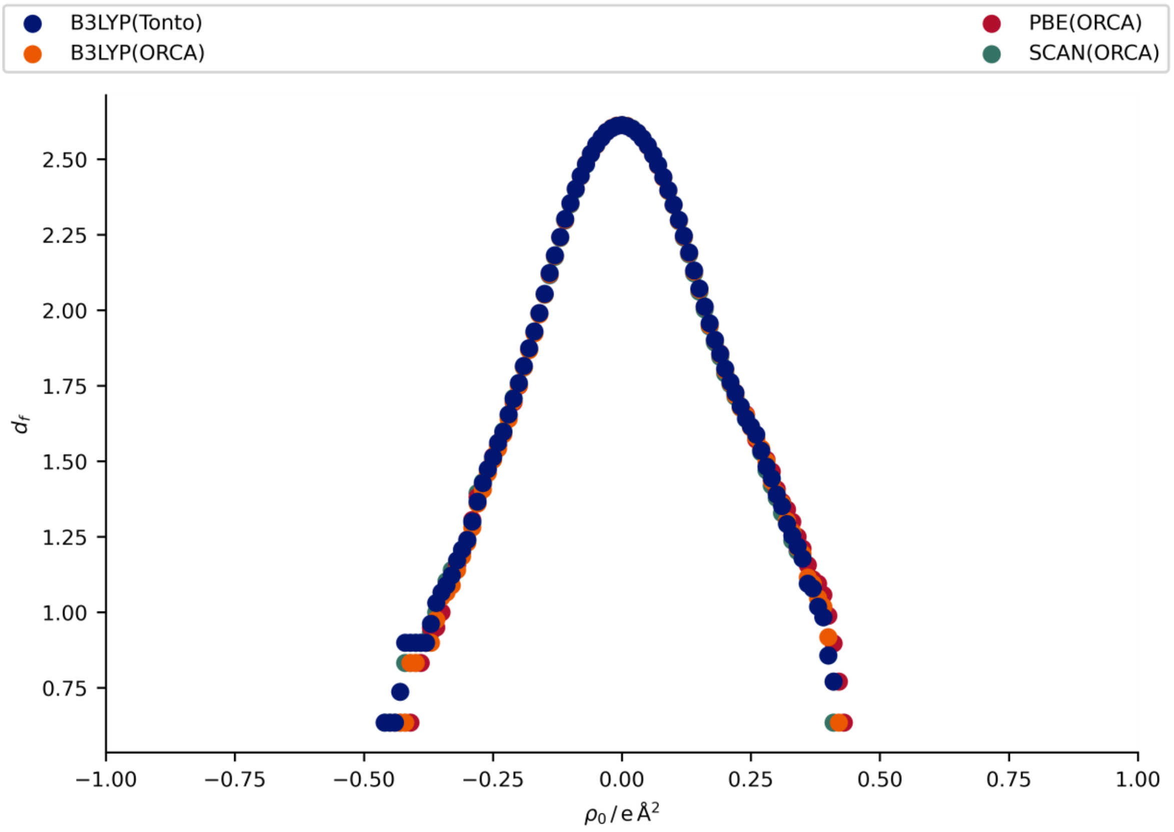

Figure S15 Henn-Meindl plots of all non-periodic calculations in this work for the 8-hydroxyquinone hydrogen maleate at 15 K (**HMa-8HQ**) dataset.

S5.3. Hexaqua Magnesium Maleate at 15 K

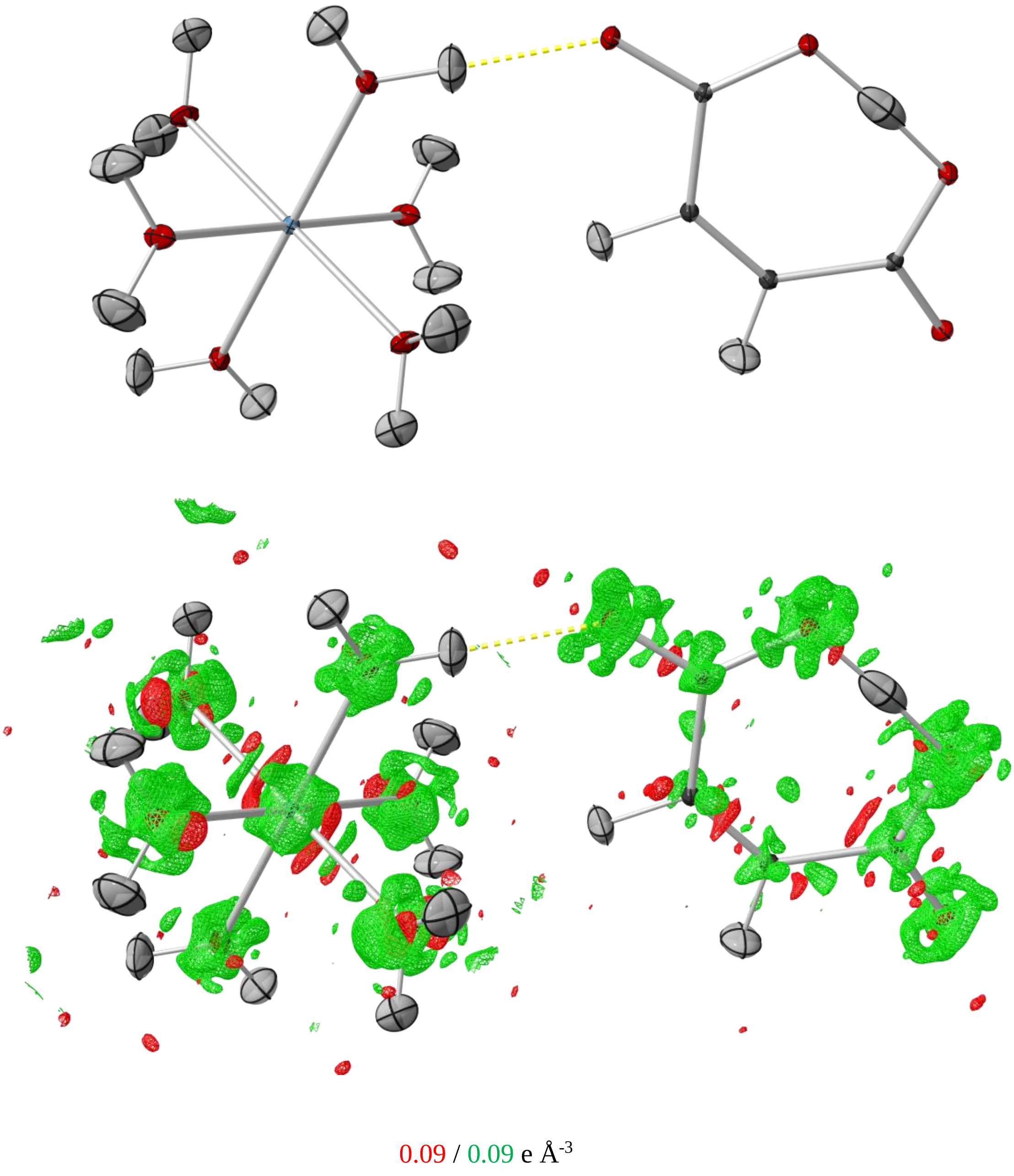

Figure S16 Resulting structure for **HMa-Mg** from the PAW-HAR refinement with the SCAN functional without (top) and with (bottom) difference electron density. All anisotropic displacement parameters are depicted at the 50 % probability level.

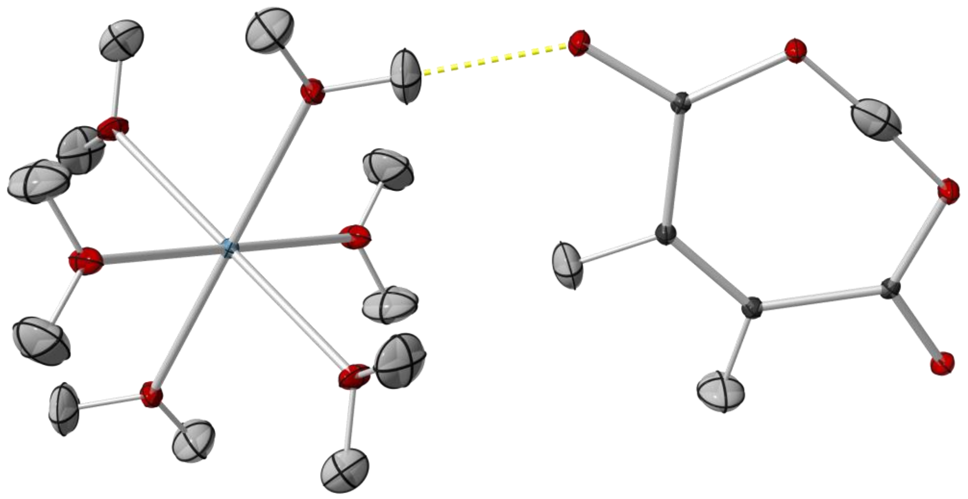

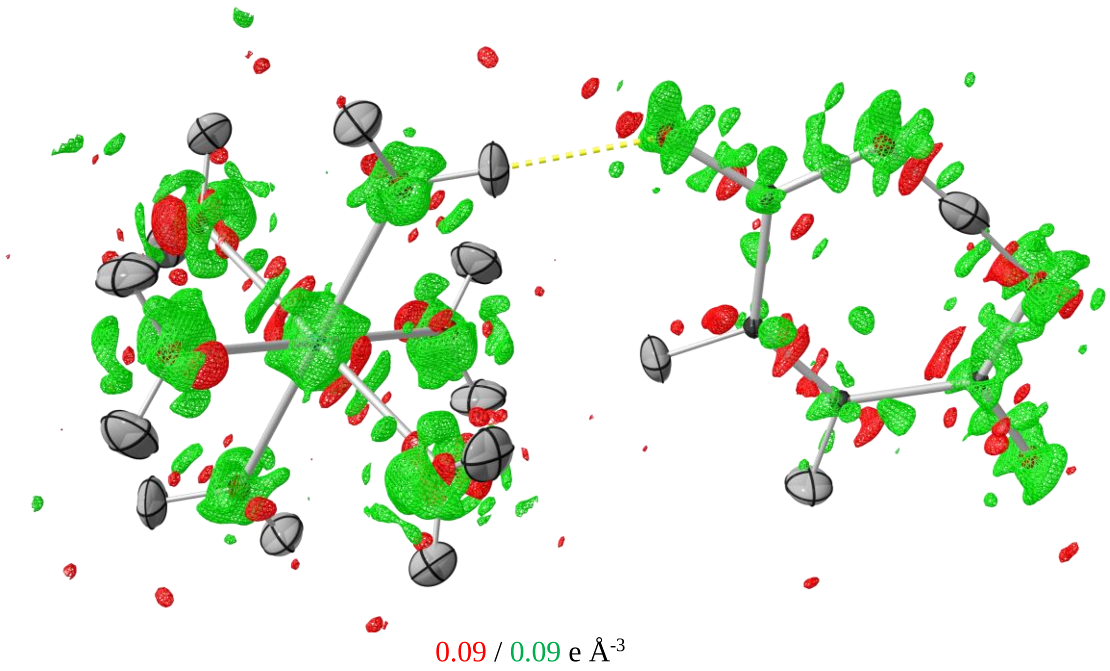

Figure S17 Resulting structure for **HMa-Mg** from the refinement with 8 Å of cluster charges in tonto without (top) and with (bottom) difference electron density. All anisotropic displacement parameters are depicted at the 50 % probability level.

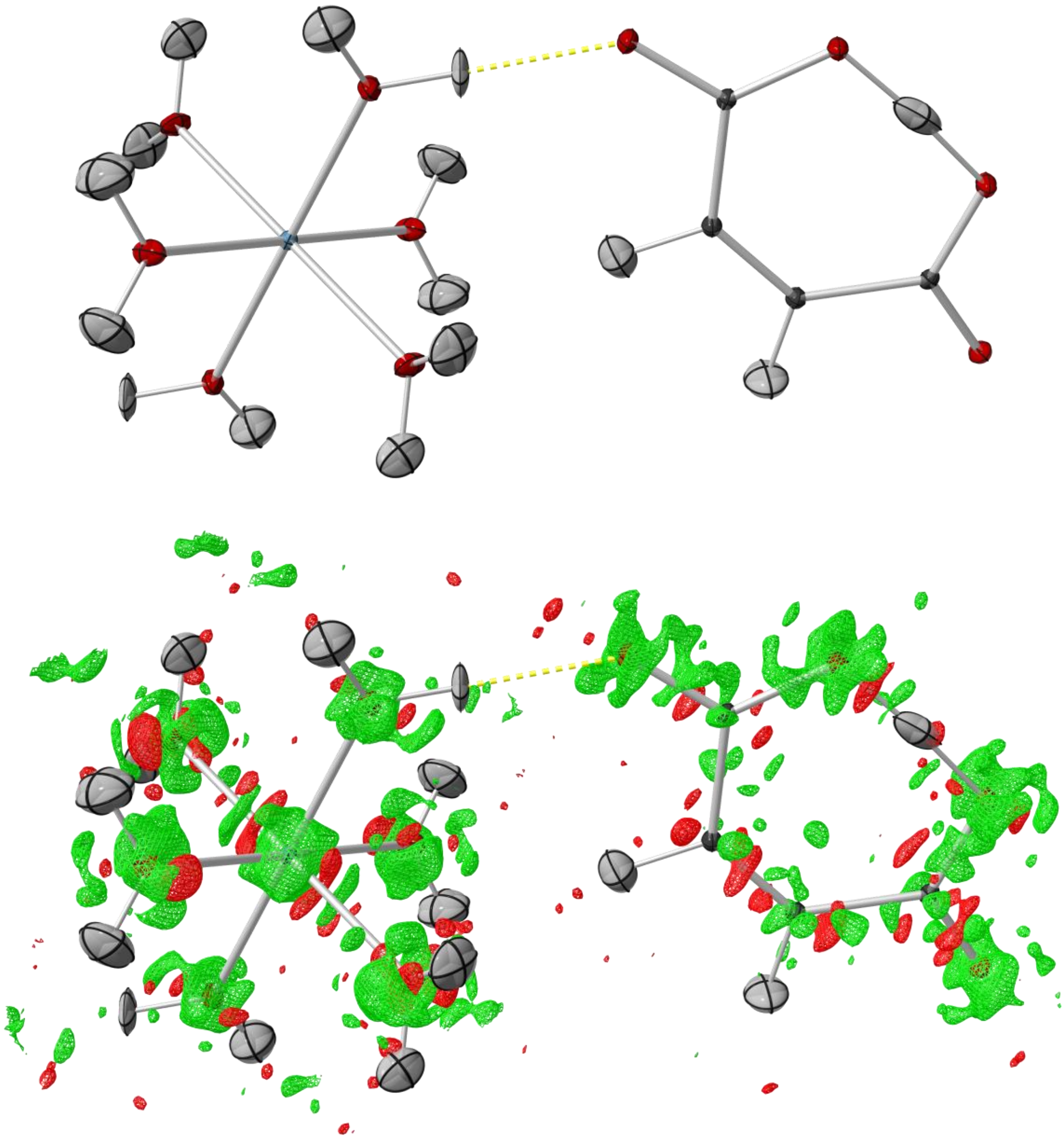

0.09 / 0.09 e Å^{-3}

Figure S18 Resulting structure for **HMa-Mg** from the refinement of the isolated molecule density calculation in Orca without (top) and with (bottom) difference electron density. All anisotropic displacement parameters are depicted at the 50 % probability level.

Table S8 Aggregated quality indicators the agreement in X-ray intensities and hydrogen atom description from for all density sources for the PAW-HAR calculations of the hexaqua magnesium hydrogen maleate at 15 K (**HMa-Mg**) dataset

functional	$wR_2(F^2)$ %	*GOF*	Δr mÅ	$\|\Delta r\|$ mÅ	*wRMSD* (Δr)	ΔU_{ij} 10^{-2} Å^2	$\|\Delta U_{ij}\|$ 10^{-2} Å^2	*wRMSD* (ΔU_{ij})	S_{12} %	V_X/V_N
PW	3.27	2.88	-6	7	1.92	-0.2	0.4	2.69	3.2	0.6
±			6	3		0.6	0.4		2.2	0.2
BLYP	3.20	2.82	-9	10	3.01	0.0	0.3	2.27	1.8	1.0
±			7	5		0.6	0.5		2.0	0.2
PW91	3.06	2.69	-8	9	2.81	-0.0	0.3	2.22	1.7	0.9
±			6	4		0.5	0.4		1.6	0.2
PBE	3.04	2.67	-7	9	2.75	-0.0	0.3	2.22	1.6	0.9
±			6	4		0.5	0.4		1.6	0.2
revPBE	2.96	2.61	-8	9	3.02	0.0	0.3	2.36	1.5	1.1
±			6	4		0.5	0.4		1.5	0.3
RPBE	2.94	2.59	-8	9	3.05	0.0	0.3	2.37	1.4	1.1
±			6	5		0.5	0.4		1.5	0.3
TPSS	3.00	2.64	-8	9	2.97	0.0	0.3	2.14	1.5	1.0
±			6	5		0.5	0.4		1.5	0.2
SCAN	2.85	2.51	-5	6	2.04	-0.1	0.3	2.21	1.4	0.8
±			4	4		0.4	0.4		1.1	0.2
revSCAN	2.78	2.45	-5	5	2.10	-0.1	0.3	2.22	1.3	0.8
±			5	4		0.4	0.3		0.9	0.2
vdW-DF	3.08	2.71	-10	11	3.38	0.1	0.4	2.33	1.6	1.2
±			7	5		0.6	0.5		1.7	0.3
vdW-DF2	3.33	2.93	-10	11	3.01	0.1	0.4	2.52	2.0	1.2
±			8	5		0.6	0.5		2.1	0.3
BEEF-vdW	3.04	2.67	-9	10	3.11	0.1	0.4	2.28	1.5	1.2
±			7	5		0.6	0.4		1.5	0.3

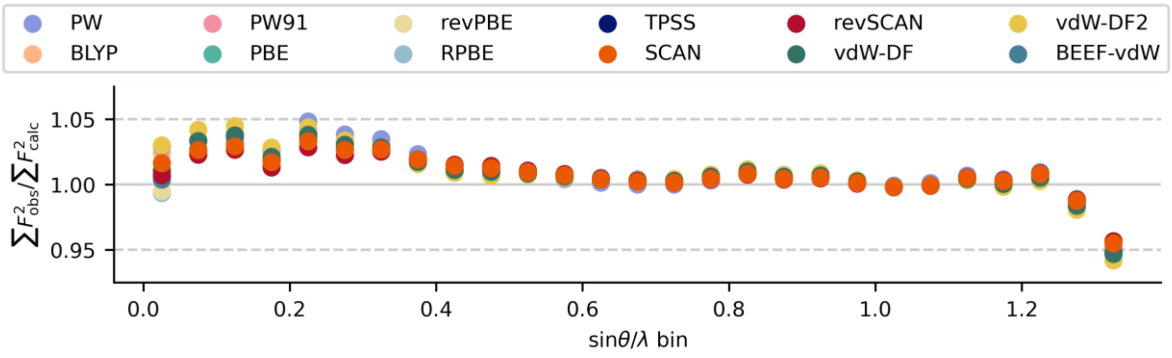

Figure S19 DRK plots of all periodic PAW calculations in this work for the hexaqua magnesium hydrogen maleate at 15 K (**HMa-Mg**) dataset.

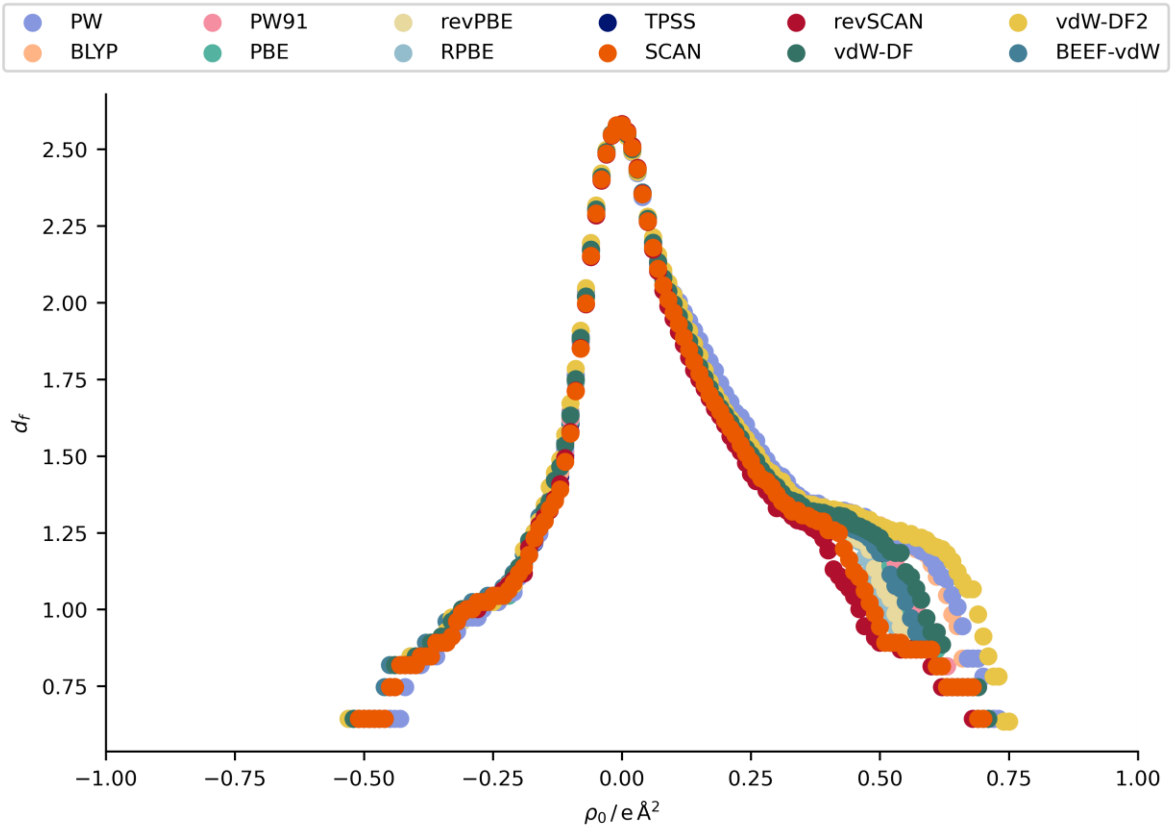

Figure S20 Henn-Meindl plots of all periodic PAW calculations in this work for the hexaqua magnesium hydrogen maleate at 15 K (**HMa-Mg**) dataset.

Table S9 Aggregated quality indicators the agreement in X-ray intensities and hydrogen atom description from for all density sources for the non-periodical calculations of the hexaqua magnesium hydrogen maleate at 15 K (**HMa-Mg**) dataset

functional	$wR_2(F^2)$ %	*GOF*	Δr mÅ	$\|\Delta r\|$ mÅ	*wRMSD* (Δr)	ΔU_{ij} 10^{-2} Å^2	$\|\Delta U_{ij}\|$ 10^{-2} Å^2	*wRMSD* (ΔU_{ij})	S_{12} %	V_X/V_N
			HAR non-periodic with 8 Å of cluster charges in Tonto							
B3LYP	2.91	2.56	-6.6	7	2.63	-0.0	0.3	2.22	1.5	1.0
±			5.9	5		0.4	0.2		0.9	0.2
			HAR non-periodic isolated molecule conditions in ORCA							
B3LYP	3.05	2.68	-12	14	4.59	-0.0	0.4	2.53	3.3	1.1
±			11	7		0.5	0.3		3.6	0.5
PBE	3.11	2.74	-12	15	4.67	-0.0	0.4	2.68	3.5	1.1
±			12	7		0.5	0.4		3.5	0.5
SCAN	2.96	2.60	-11	14	4.81	-0.1	0.4	2.66	4.0	1.0
±			11	8		0.5	0.3		5.8	0.5

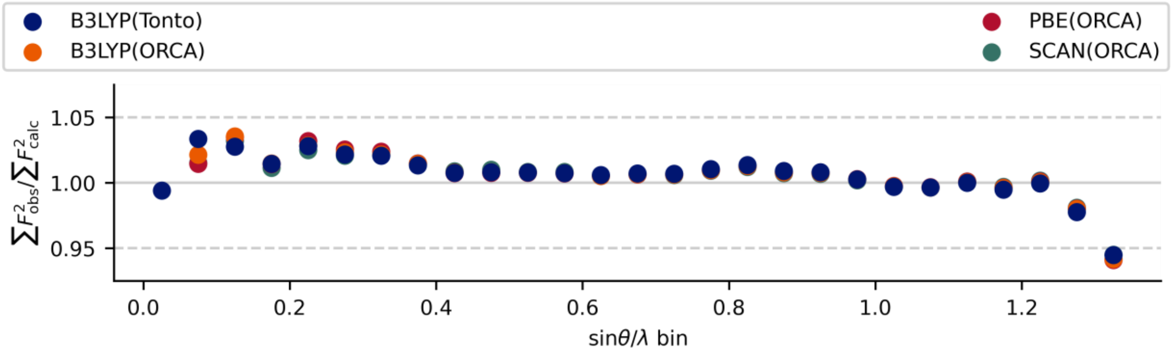

Figure S21 DRK plots of all non-periodic calculations in this work for the hexaquamagnesium hydrogen maleate at 15 K (**HMa-Mg**) dataset.

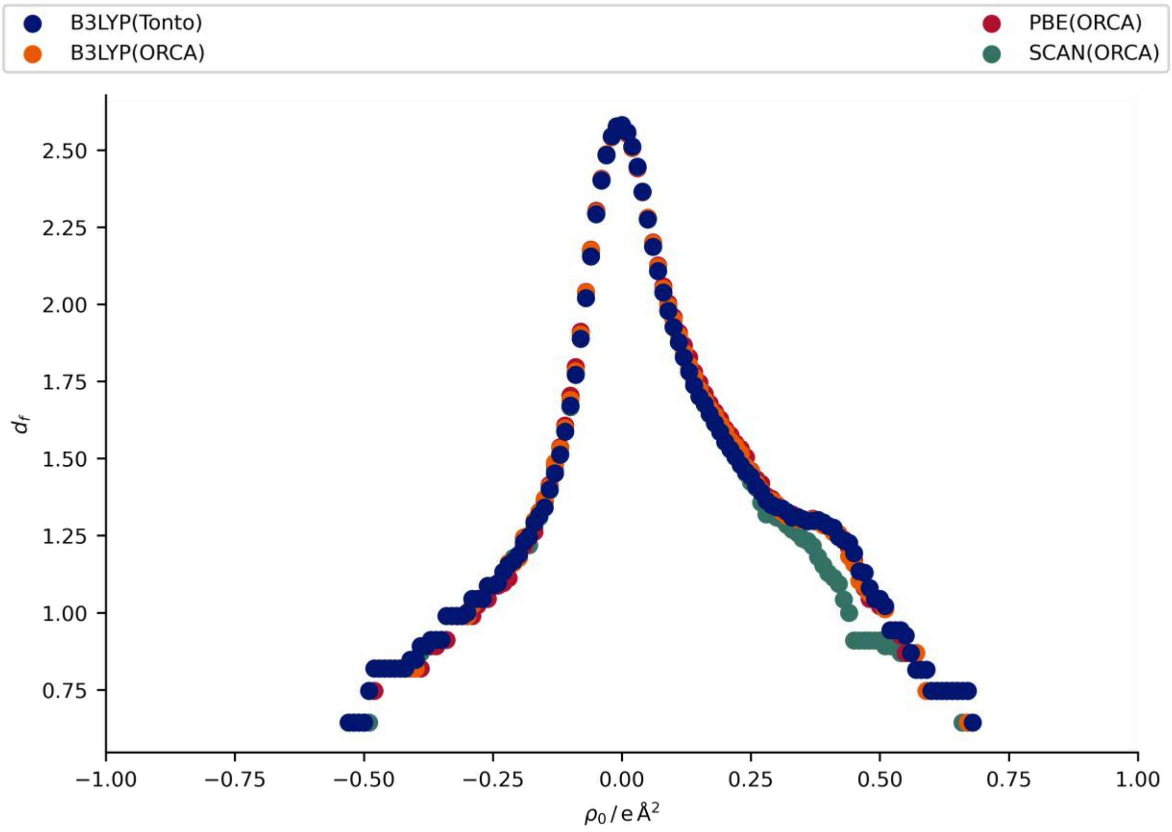

Figure S22 Henn-Meindl plots of all non-periodic calculations in this work for the hexaqua magnesium hydrogen maleate at 15 K (**HMa-Mg**) dataset.

S5.4. Xylitol at 122 K

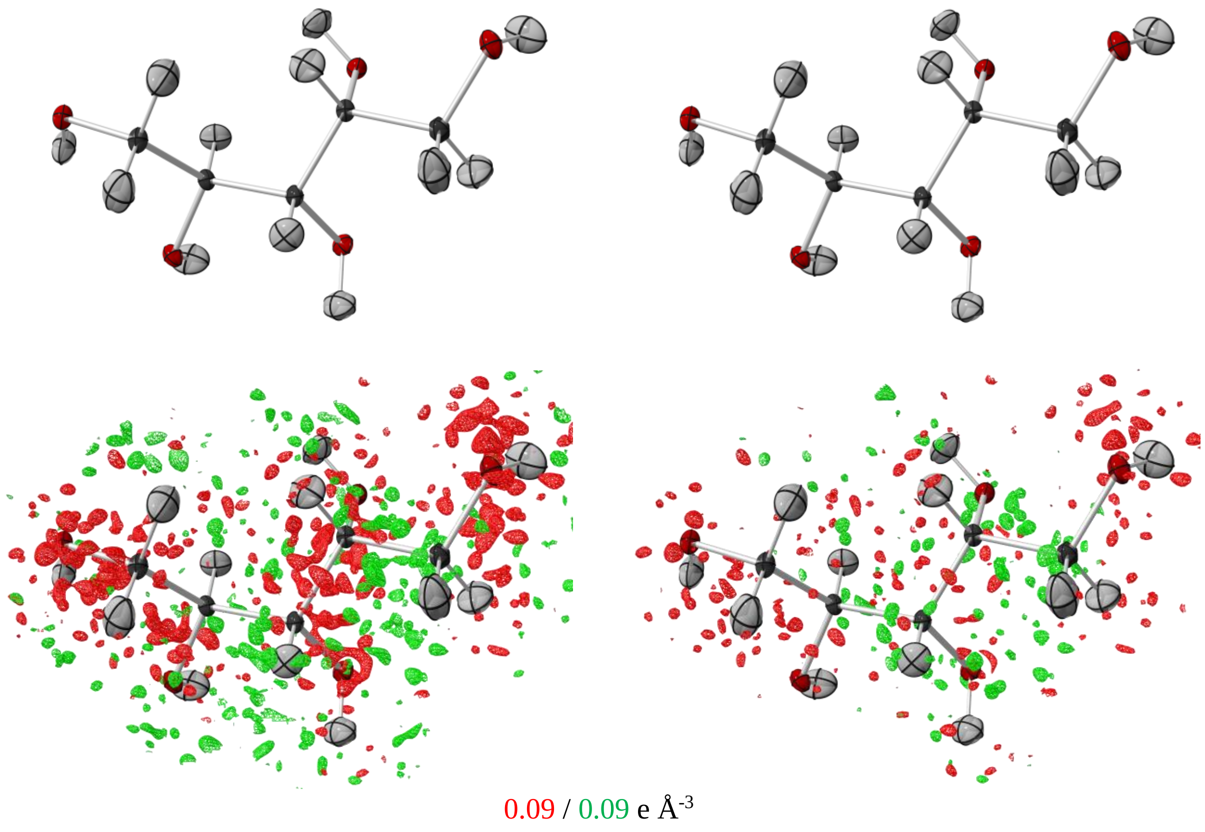

Figure S23 Structure images for **Xy** from the PAW-HAR refinement with the SCAN functional: Left: Resulting structure without the refinement of an extinction parameter. Right: Resulting structure of the refinement with an extinction parameter. All anisotropic displacement parameters are depicted at the 50 % probability level. For each structure there is an image without (top) and with (bottom) difference electron density.

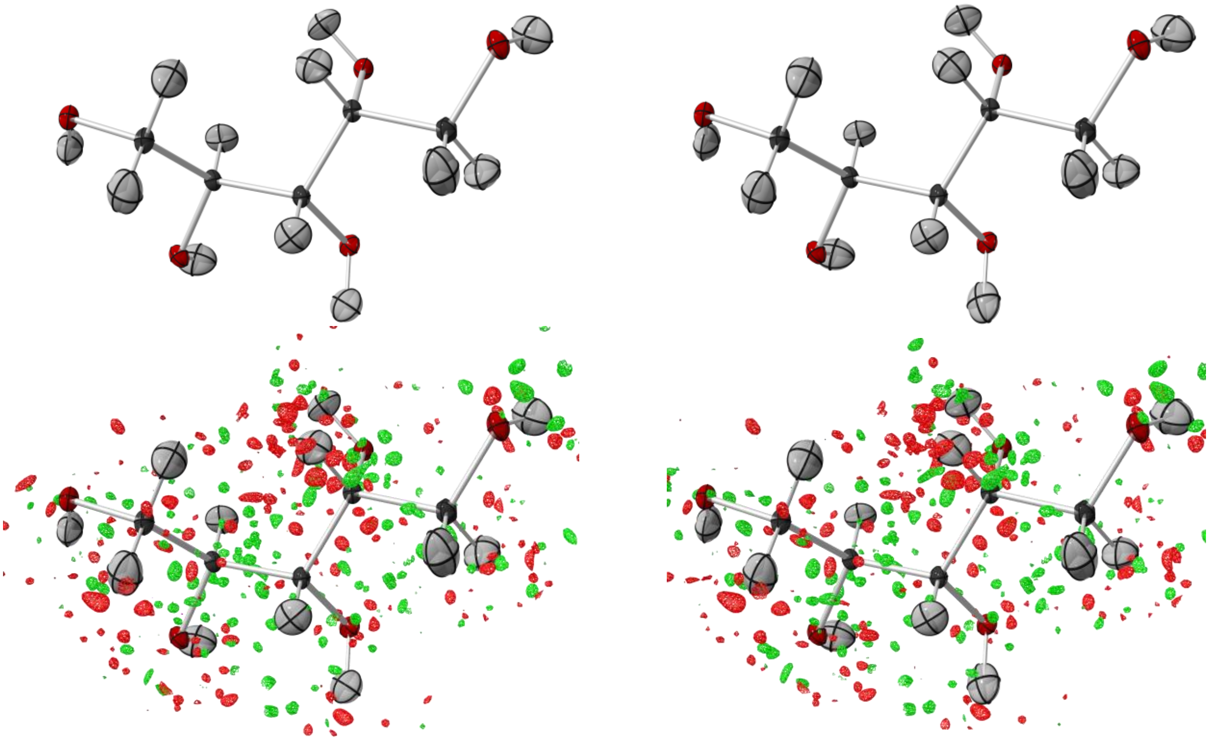

0.09 / 0.09 e Å^{-3}

Figure S24 Structure images for **Xy** with extinction refinement: Left: Resulting structure from the refinement with 8 Å of cluster charges in tonto. Right: Resulting structure of the refinement of the isolated molecule density calculation in Orca. All anisotropic displacement parameters are depicted at the 50 % probability level. For each structure there is an image without (top) and with (bottom) difference electron density.

Table S10 Aggregated quality indicators the agreement in X-ray intensities and hydrogen atom description from for all density sources for the PAW-HAR calculations of the xylitol at 122 K (**Xy**) dataset

functional	$wR_2(F^2)$	*GOF*	Δr	$\lvert\Delta r\rvert$	*wRMSD*	ΔU_{ij}	$\lvert\Delta U_{ij}\rvert$	*wRMSD*	S_{12}	V_X/V_N
	%		mÅ	mÅ	(Δr)	10^{-2} Å^2	10^{-2} Å^2	(ΔU_{ij})	%	
PW	2.35	0.75	-14	15	3.45	-0.1	0.6	3.54	6.5	0.8
±			12	11		0.8	0.5		4.7	0.3
BLYP	2.24	0.71	-16	16	3.95	0.2	0.6	3.57	5.8	1.5
±			12	12		0.7	0.5		4.3	0.3
PW91	2.23	0.71	-14	14	3.60	0.1	0.5	3.47	5.5	1.2
±			11	11		0.7	0.5		4.2	0.3
PBE	2.22	0.70	-14	14	3.55	0.1	0.5	3.45	5.5	1.3

±			11	11		0.7	0.5		4.2	0.3
revPBE	2.19	0.69	-14	14	3.44	0.2	0.6	3.49	5.4	1.4
±			10	10		0.7	0.5		4.1	0.3
RPBE	2.18	0.69	-14	14	3.45	0.2	0.6	3.50	5.4	1.5
±			10	10		0.7	0.5		4.1	0.3
TPSS	2.21	0.70	-14	14	3.55	0.2	0.6	3.54	5.5	1.4
±			11	11		0.7	0.5		4.1	0.3
SCAN	2.14	0.68	-9	11	2.67	0.1	0.5	3.46	5.3	1.2
±			9	8		0.7	0.5		4.2	0.2
revSCAN	2.12	0.67	-8	9	2.45	0.1	0.5	3.44	5.2	1.2
±			8	7		0.7	0.5		4.3	0.2
vdW-DF	2.20	0.70	-17	17	4.00	0.3	0.6	3.71	5.7	1.7
±			11	11		0.8	0.5		4.2	0.3
vdW-DF2	2.26	0.72	-18	18	4.25	0.3	0.7	3.75	6.0	1.7
±			13	13		0.8	0.5		4.3	0.4
BEEF-vdW	2.20	0.70	-14	14	3.53	0.3	0.6	3.69	5.7	1.7
±			10	10		0.8	0.5		4.2	0.3

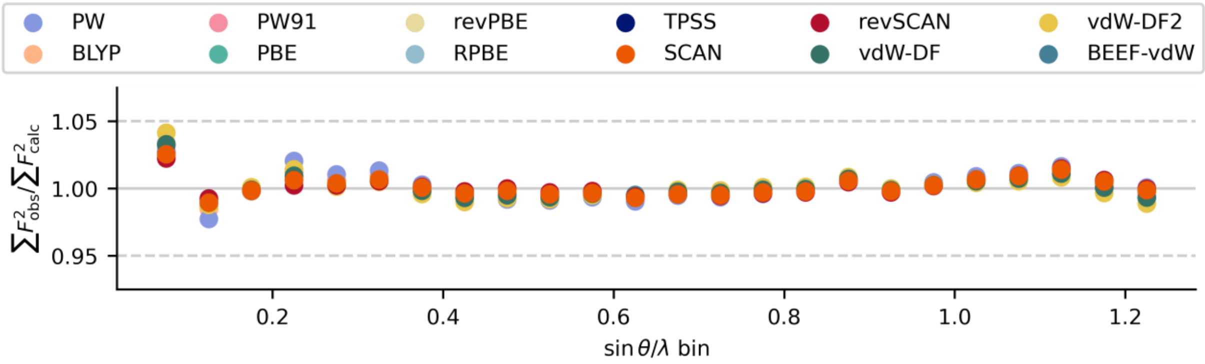

Figure S25DRK plots of all periodic PAW calculations in this work for the xylitol at 122 K (**Xy**) dataset.

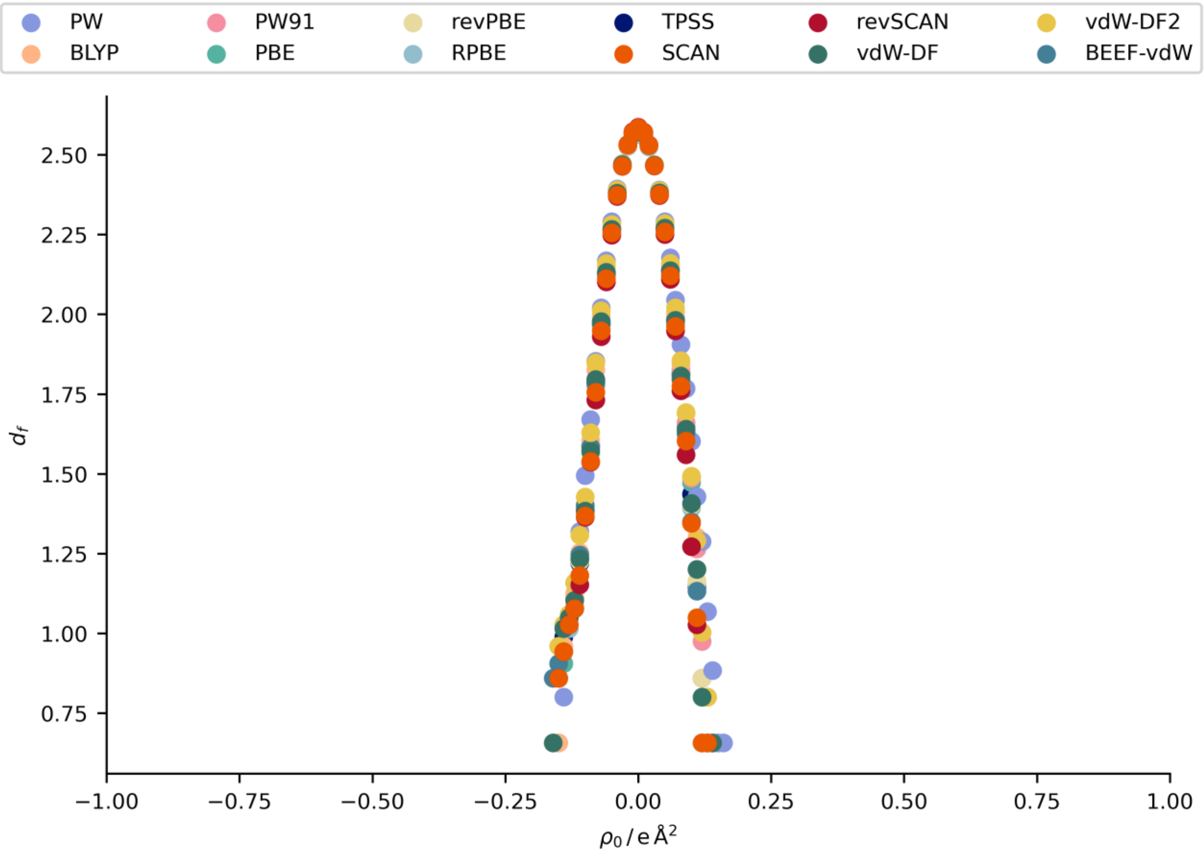

Figure S26 Henn-Meindl plots of all periodic PAW calculations in this work for the xylitol at 122 K (**Xy**) dataset.

Table S11 Aggregated quality indicators the agreement in X-ray intensities and hydrogen atom description from for all density sources for the non-periodical calculations of the xylitol at 122 K (**Xy**) dataset

functional	$wR_2(F^2)$ %	*GOF*	Δr mÅ	$\lvert\Delta r\rvert$ mÅ	*wRMSD* (Δr)	ΔU_{ij} 10^{-2} Å^2	$\lvert\Delta U_{ij}\rvert$ 10^{-2} Å^2	*wRMSD* (ΔU_{ij})	S_{12} %	V_X/V_N
			HAR non-periodic with 8 Å of cluster charges in Tonto							
B3LYP	2.12	0.67	-15	15	3.96	0.2	0.6	3.68	5.5	1.7
±			11	11		0.7	0.5		4.0	0.3
			HAR non-periodic isolated molecule conditions in ORCA							
B3LYP	2.18	0.69	-22	22	5.26	0.3	0.7	3.78	6.2	1.8
±			15	15		0.8	0.5		3.3	0.6
PBE	2.22	0.70	-24	24	5.52	0.2	0.7	3.80	6.4	1.7
±			15	15		0.8	0.5		3.2	0.6
SCAN	2.14	0.68	-20	20	4.94	0.2	0.6	3.72	5.9	1.7
±			14	14		0.8	0.5		3.2	0.5

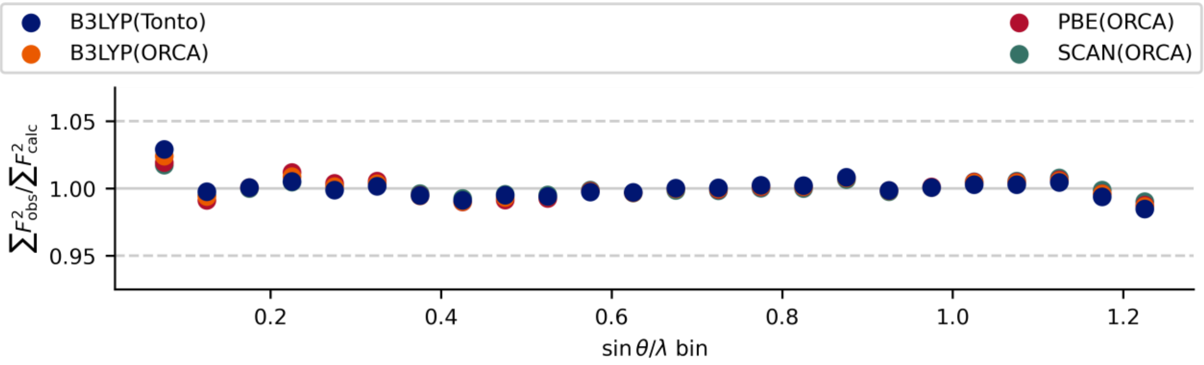

Figure S27 DRK plots of all non-periodic calculations in this work for the xylitol at 122 K (**Xy**) dataset.

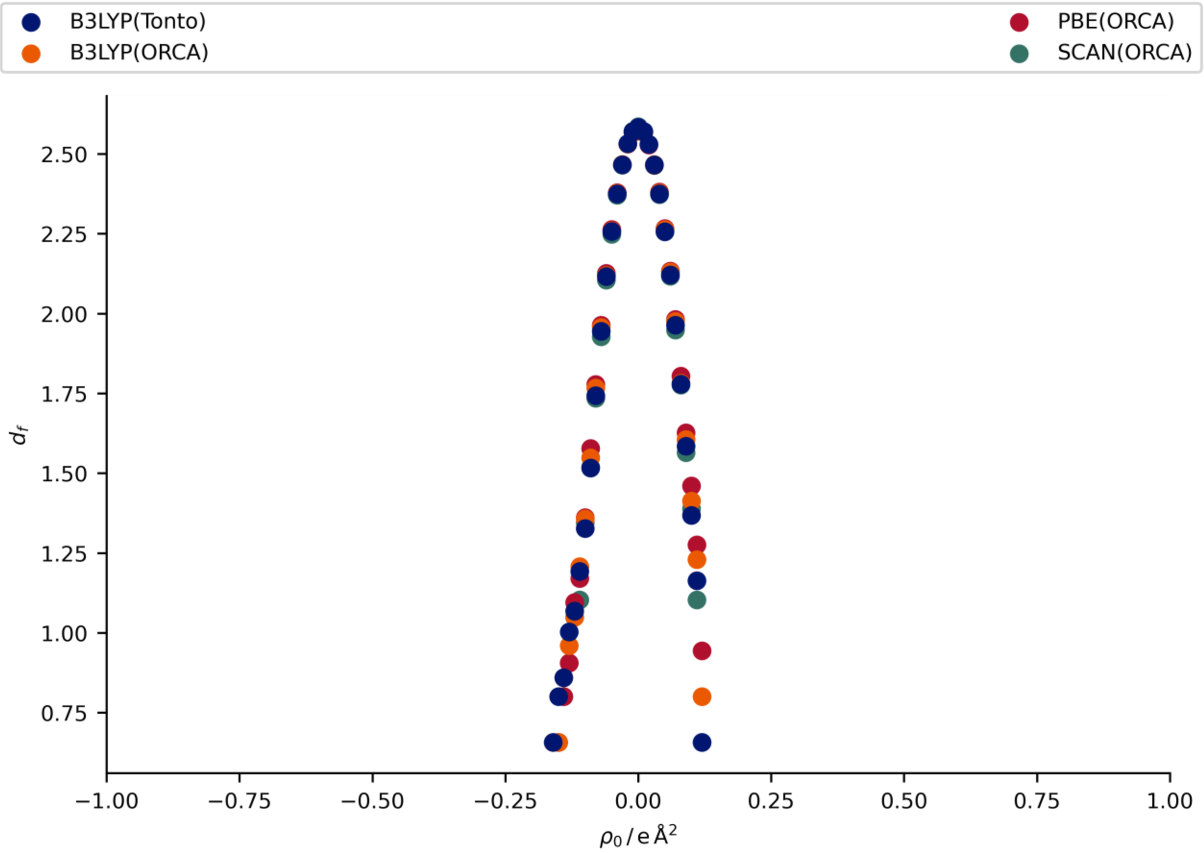

Figure S28 Henn-Meindl plots of all non-periodic calculations in this work for the xylitol at 122 K (**Xy**) dataset.

Table S12 Aggregated quality indicators the agreement in X-ray intensities and hydrogen atom description for the SCAN PAW-HAR calculations of the **Xy** dataset with and without extinction correction.

Extinction	$wR_2(F^2)$	*GOF*	Δr	$\|\Delta r\|$	*wRMSD*	ΔU_{ij}	$\|\Delta U_{ij}\|$	*wRMSD*	S_{12}	V_X/V_N

	%		mÅ	mÅ	(Δr)	10^{-2} Å^2	10^{-2} Å^2	(ΔU_{ij})	%	
Yes	2.14	0.68	-9	11	2.67	0.1	0.5	3.46	5.3	1.2
±			9	8		0.7	0.5		4.2	0.2
No	2.28	0.73	-5	9	2.25	0.1	0.6	3.41	5.5	1.1
±			10	6		0.7	0.5		4.3	0.2

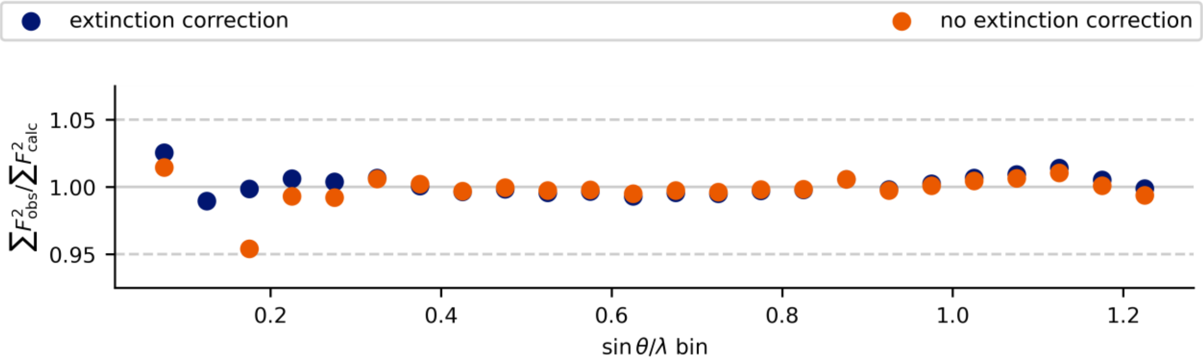

Figure S29DRK plots of the SCAN PAW calculations of the **Xy** dataset with and without extinction correction.

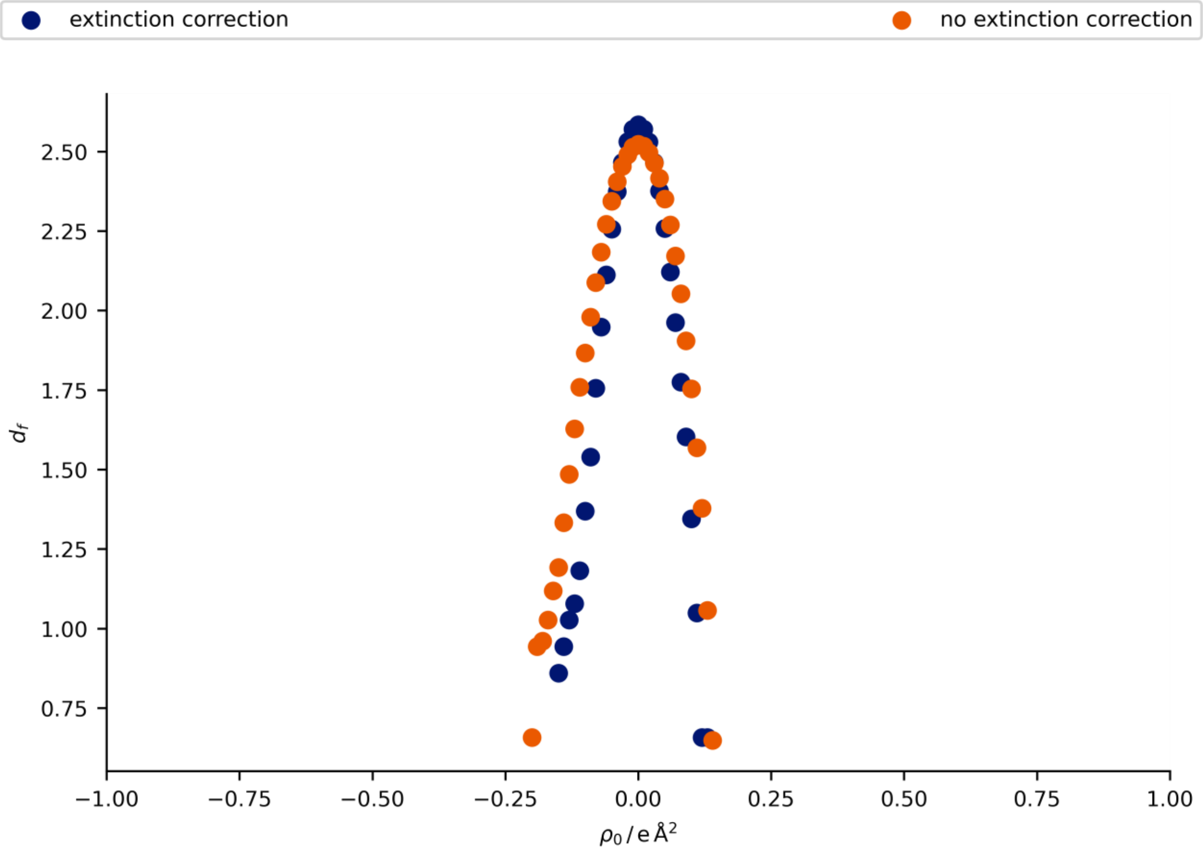

Figure S30 Henn-Meindl plots of the SCAN PAW calculations of the **Xy** dataset with and without extinction correction.

S5.5. Urea at 123 K

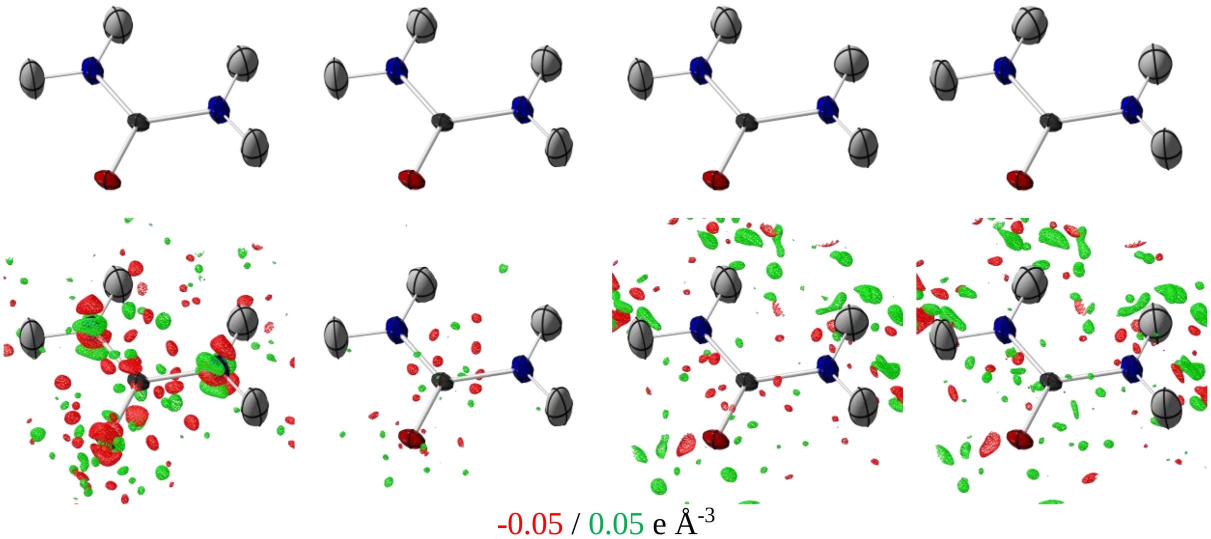

Figure S31 Structure images for **Urea**: Outer Left: Resulting structure from the PAW-HAR refinement with the SCAN functional. Centre Left: Resulting structure from the PAW-HAR refinement with the SCAN functional and refined Gram-Charlier parameters. Centre Right: Resulting structure from the refinement with 8 Å of cluster charges in tonto. Outer Right: Resulting structure of the refinement of the isolated molecule density calculation in Orca. All anisotropic displacement parameters are depicted at the 50 % probability level. For each structure there is an image without (top) and with (bottom) difference electron density.

Table S13 Aggregated quality indicators the agreement in X-ray intensities and hydrogen atom description from for all density sources for the PAW-HAR calculations of the **Urea** at 123 K dataset

functional	$wR_2(F^2)$	*GOF*	Δr	$\|\Delta r\|$	*wRMSD*	ΔU_{ij}	$\|\Delta U_{ij}\|$	*wRMSD*	S_{12}	V_X/V_N
	%		mÅ	mÅ	(Δr)	10^{-2} Å^2	10^{-2} Å^2	(ΔU_{ij})	%	
PW	1.97	2.10	-1	5	1.34	-0.1	0.6	3.51	4.7	0.8
±			5	1		0.7	0.3		1.1	0.3
BLYP	1.89	2.03	-6	6	2.02	0.1	0.6	3.66	2.8	1.5
±			3	3		0.8	0.5		1.3	0.5
PW91	1.85	1.98	-3	3	1.19	0.1	0.6	3.49	2.8	1.4
±			3	3		0.7	0.4		0.8	0.4
PBE	1.83	1.95	-2	4	1.24	0.1	0.6	3.53	2.8	1.4
±			4	2		0.7	0.4		0.9	0.4
revPBE	1.80	1.92	-1	3	1.06	0.2	0.6	3.76	2.7	1.6
±			3	1		0.7	0.5		1.2	0.5

RPBE	1.79	1.91	-1	3	1.06	0.2	0.6	3.81	2.7	1.7
±			3	1		0.8	0.5		1.2	0.5
TPSS	1.80	1.93	-4	4	1.39	0.2	0.6	3.74	2.5	1.6
±			3	3		0.8	0.5		1.3	0.5
SCAN	1.76	1.88	1	2	0.62	0.1	0.5	3.19	1.8	1.3
±			2	1		0.6	0.4		0.9	0.3
revSCAN	1.77	1.89	4	4	1.26	0.1	0.5	3.10	1.5	1.3
±			0	0		0.6	0.4		0.7	0.3
vdW-DF	1.82	1.96	-8	8	2.37	0.2	0.7	3.92	2.6	1.8
±			3	3		0.8	0.5		1.6	0.5
vdW-DF2	1.97	2.13	-13	13	3.69	0.2	0.7	3.70	2.7	1.7
±			3	3		0.8	0.5		1.7	0.5
BEEF-vdW	1.80	1.94	-5	5	1.62	0.2	0.7	3.99	2.4	1.8
±			3	3		0.8	0.5		1.5	0.5

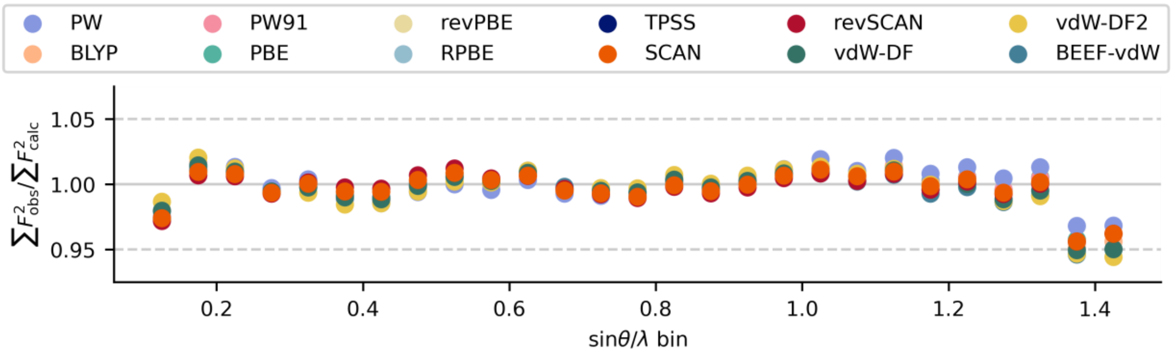

Figure S32 DRK plots of all periodic PAW calculations in this work for the **Urea** at 123 K dataset.

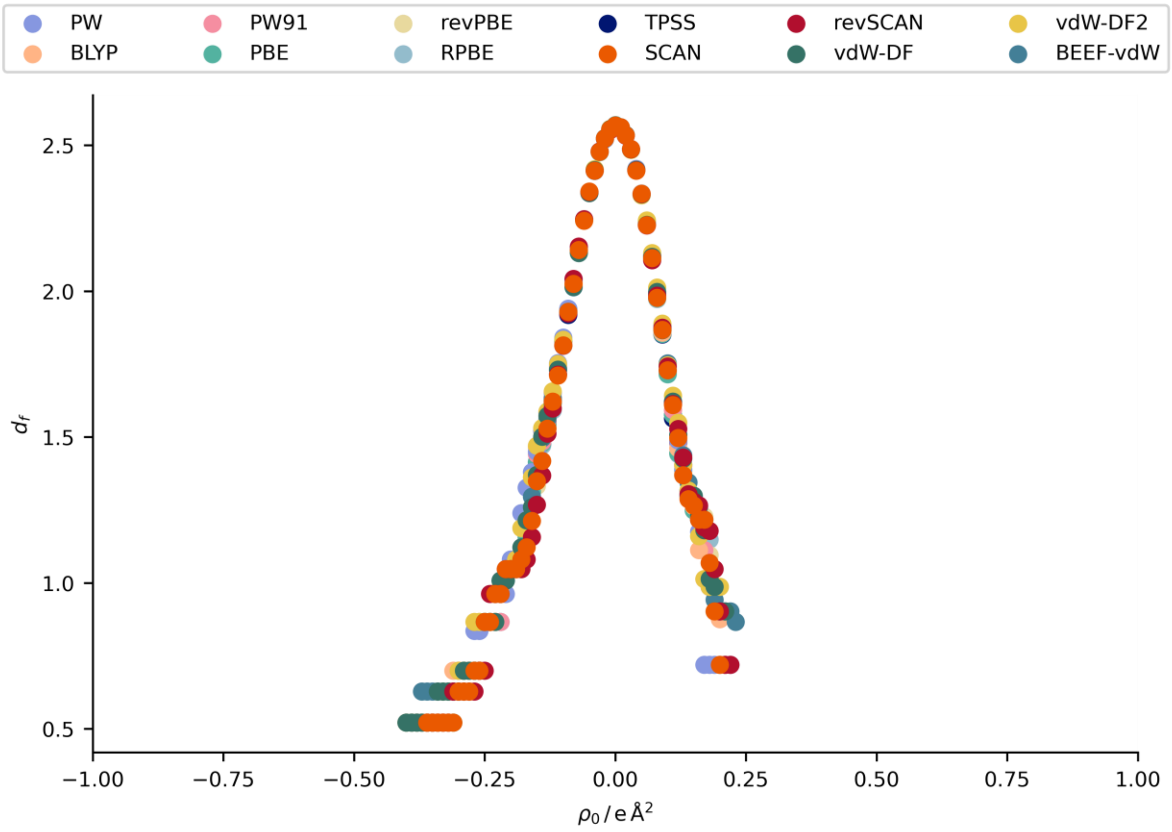

Figure S33 Henn-Meindl plots of all periodic PAW calculations in this work for the **Urea** at 123 K dataset.

Table S14 Aggregated quality indicators the agreement in X-ray intensities and hydrogen atom description from for all density sources for the non-periodical calculations of the **Urea** at 123 K dataset

functional	$wR_2(F^2)$	*GOF*	Δr	$\lvert\Delta r\rvert$	*wRMSD*	ΔU_{ij}	$\lvert\Delta U_{ij}\rvert$	*wRMSD*	S_{12}	V_X/V_N
	%		mÅ	mÅ	(Δr)	10^{-2} Å^2	10^{-2} Å^2	(ΔU_{ij})	%	
HAR non-periodic with 8 Å of cluster charges in Tonto										
B3LYP	1.80	1.93	-7.0	7	2.02	0.1	0.6	3.73	1.8	1.6
±			2.0	2		0.7	0.5		1.0	0.3
HAR non-periodic isolated molecule conditions in ORCA										
B3LYP	2.16	2.31	-3	9	2.14	0.3	0.7	4.57	3.2	2.0
±			9	3		1.0	0.8		1.5	0.8
PBE	2.20	2.36	-1	7	1.60	0.4	0.8	4.78	4.2	1.9
±			7	1		1.0	0.8		1.3	0.8
SCAN	2.11	2.26	1	10	2.42	0.4	0.7	4.64	3.0	2.1
±			10	1		1.0	0.8		1.4	0.8

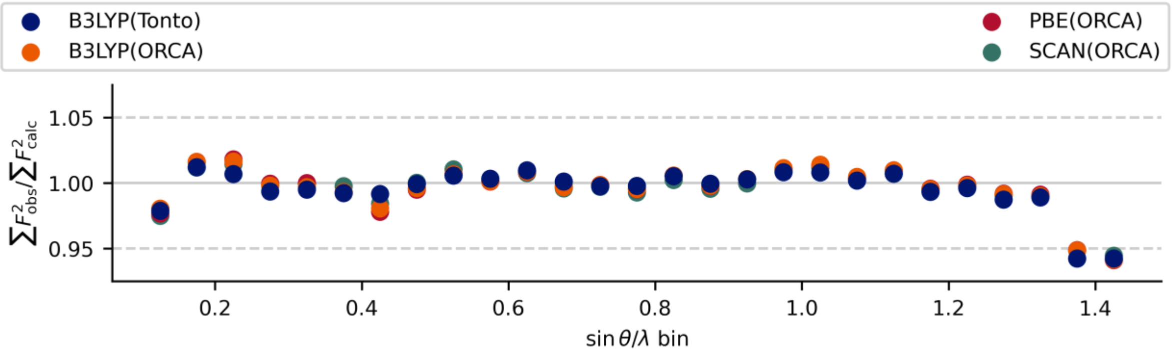

Figure S34 DRK plots of all non-periodic calculations in this work for the **Urea** at 123 K dataset.

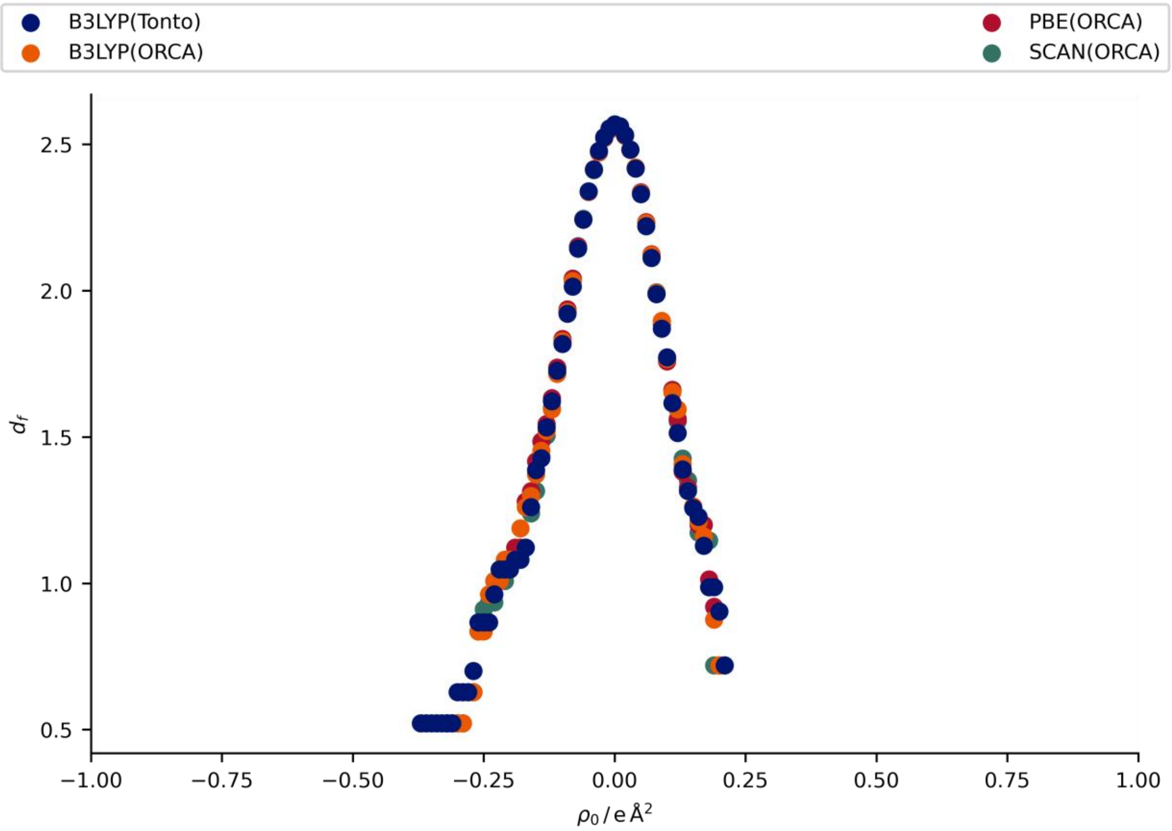

Figure S35 Henn-Meindl plots of all non-periodic calculations in this work for the **Urea** at 123 K dataset.

S6. Gram-Charlier refinement of urea.

In Section 3.3.4 of the publication, we postulated that the improvement in the density description of **Urea** reveals the difference electron density pattern indicating anharmonic vibration, which we then claimed to have successfully described with refinement of Gram-Charlier parameter refinement up to the fourth order. Here we want to describe our approach to check the correct refinement within our

script and to then validate the Gram-Charlier description and finally want to give the resulting quality indicators.

S6.1. Validation against Olex Refine

Olex (Dolomanov *et al.*, 2009) provides the possibility to read in pre-written .tsc files (Midgley *et al.*, 20.11.2019) for use in *olex.refine*. At the same time, we can also refine Gram-Charlier parameters up to the fourth order. In order to validate the performance of our own script we first did a refinement until convergence. From the final positions we then calculated atomic form factors with GPAW and the SCAN functional used for refinement in our script and wrote a .tsc file. This was then used for one refinement in Olex. The resulting Gram-Charlier parameters can be found in Table S15 for the Third order Gram-Charlier parameter and in Table S16 and Table S17 for the fourth order. As third order Gram-Charlier refinement of carbon did not yield values, that were significantly different from zero, no Gram-Charlier parameters for carbon were refined in the calculation.

Obtained values are not identical but differences are well below the estimated standard deviation of the obtained parameters. Therefore, we the refinement of Gram-Charlier parameters in our script was validated.

Table S15 Resulting third order Gram-Charlier parameters refined with our script and refined against a .tsc file for the final coordinates from our script in NoSpherA2. All values have to be multiplied by 10^{-6}. Values marked with zero without an estimated standard deviation were not refined due to symmetry constraints.

Atom	Refinement	c_{111}/c_{222}	c_{333}	c_{112}/c_{122}	c_{113}/c_{223}	c_{133}/c_{233}	c_{123}
O	Our Script	0	-0.16(9)	0	-0.33(5)	0	0.07(13)
	NoSpherA2	0	-0.14(10)	0	-0.32(7)	0	0.08(11)
N	Our Script	-1.41(14)	0.44(11)	0.45(6)	1.41(8)	0.01(5)	-1.44(13)
	NoSpherA2	-1.39(14)	0.45(11)	0.46(6)	1.42(8)	0.02(5)	-1.44(13)

Table S16 First part of the resulting fourth order Gram-Charlier parameters refined with our script and refined against a .tsc file for the final coordinates from our script in NoSpherA2. All values have to be multiplied by 10^{-9}. Values marked with zero without an estimated standard deviation were not refined due to symmetry constraints.

Atom	Refinement	d_{1111}/d_{2222}	d_{3333}	d_{1112}/d_{1222}	d_{1113}/d_{2223}	d_{1333}/d_{2333}
O	Our Script	31(8)	23(9)	7(6)	0	0

	NoSpherA2	30(10)	23(12)	7(8)	0	0
N	Our Script	-21(12)	-25(10)	72(9)	-3(7)	-1(4)
	NoSpherA2	-17(12)	-24(10)	72(9)	-2(7)	0(4)

Table S17 Second part of the resulting fourth order Gram-Charlier parameters refined with our script and refined against a .tsc file for the final coordinates from our script in NoSpherA2. All values have to be multiplied by 10^{-9}. Values marked with zero without an estimated standard deviation were not refined due to symmetry constraints.

Name		d_{1122}	d_{1133}/d_{2233}	d_{1123}/d_{1223}	d_{1233}
O	Our Script	-2(5)	2(3)	0	-4(5)
	NoSpherA2	-3(7)	2(4)	0	-4(5)
N	Our Script	-81(10)	8(4)	6(3)	-1(5)
	NoSpherA2	-80(10)	8(4)	6(3)	-1(5)

S6.2. Significance, positive probability density and Kuhs' rule

Additionally, we can see that for both refined atoms there are third and fourth order Gram-Charlier parameters with a value of more than three estimated standard deviations. Therefore, the first criterion for the Gram-Charlier refinement is met.

For checking Kuhs' rule (Kuhs, 1992)and excluding the possibility of negative probability density from the Gram-Charlier refinement, we wrote a xd parameter file and subsequently used XDPDF from the XD2016 Suite (Volkov *et al.*, 2016) for validation. All probability density is positive. With a given $\frac{\sin(\theta)}{\lambda}$ value of 1.405 Å^{-1} we are below the required value for the refinement of fourth order Gram-Charlier parameters for the carbon atom (1.43 Å^{-1}) and well above the values for the fourth order Gram-Charlier refinement of nitrogen (1.12 Å^{-1}) and oxygen (1.28 Å^{-1}). Subsequently, we tried refining third-order Gram-Charlier parameters for carbon and third and fourth-order Gram-Charlier parameters for nitrogen and oxygen. Both oxygen and nitrogen have anharmonic vibration parameters of third and fourth order, which are significant ($|c|/\sigma(c) > 3$). However, no cijk-parameter of the carbon atom was significant. Therefore Gram-Charlier correction was only refined for the nitrogen and oxygen atoms.

S6.3. Quality indicators for the Gram-Charlier Refinement

Table S18 Aggregated quality indicators the agreement in X-ray intensities and hydrogen atom description refinements with Gram-Charlier refinement of the **Urea** dataset.

order	$wR_2(F^2)$	*GOF*	Δr	$\|\Delta r\|$	*wRMSD*	ΔU_{ij}	$\|\Delta U_{ij}\|$	*wRMSD*	S_{12}	V_X/V_N
	%		mÅ	mÅ	(Δr)	10^{-2} Å^2	10^{-2} Å^2	(ΔU_{ij})	%	
			Periodic SCAN calculation with Projector Augmented Wave HAR							
2	1.76	1.88	1.0	2.0	0.62	0.1	0.5	3.19	1.8	1.3
±			2.0	1.0		0.6	0.4		0.9	0.3
3	1.41	1.52	-1.5	1.5	0.54	0.1	0.4	3.40	1.5	1.4
±			0.5	0.5		0.6	0.5		0.4	0.3
4	1.33	1.44	1.5	1.5	0.47	0.1	0.5	3.62	1.5	1.3
±			0.5	0.5		0.7	0.5		0.3	0.3
			HAR non-periodic B3LYP with 8 Å of cluster charges in Tonto							
2	1.80	1.93	-7.0	7	2.02	0.1	0.6	3.73	1.8	1.6
±			2.0	2		0.7	0.5		1.0	0.3
4	1.41	1.53	-8.5	8.5	2.85	0.1	0.6	4.07	1.7	1.6
±			1.5	1.5		0.7	0.5		0.6	0.3

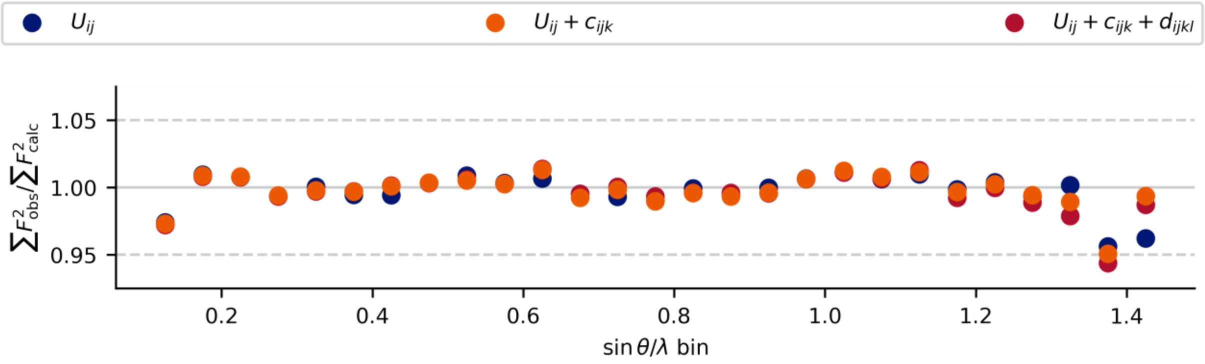

Figure S36DRK plots of the SCAN PAW calculations with Gram-Charlier refinement of the **Urea** dataset.

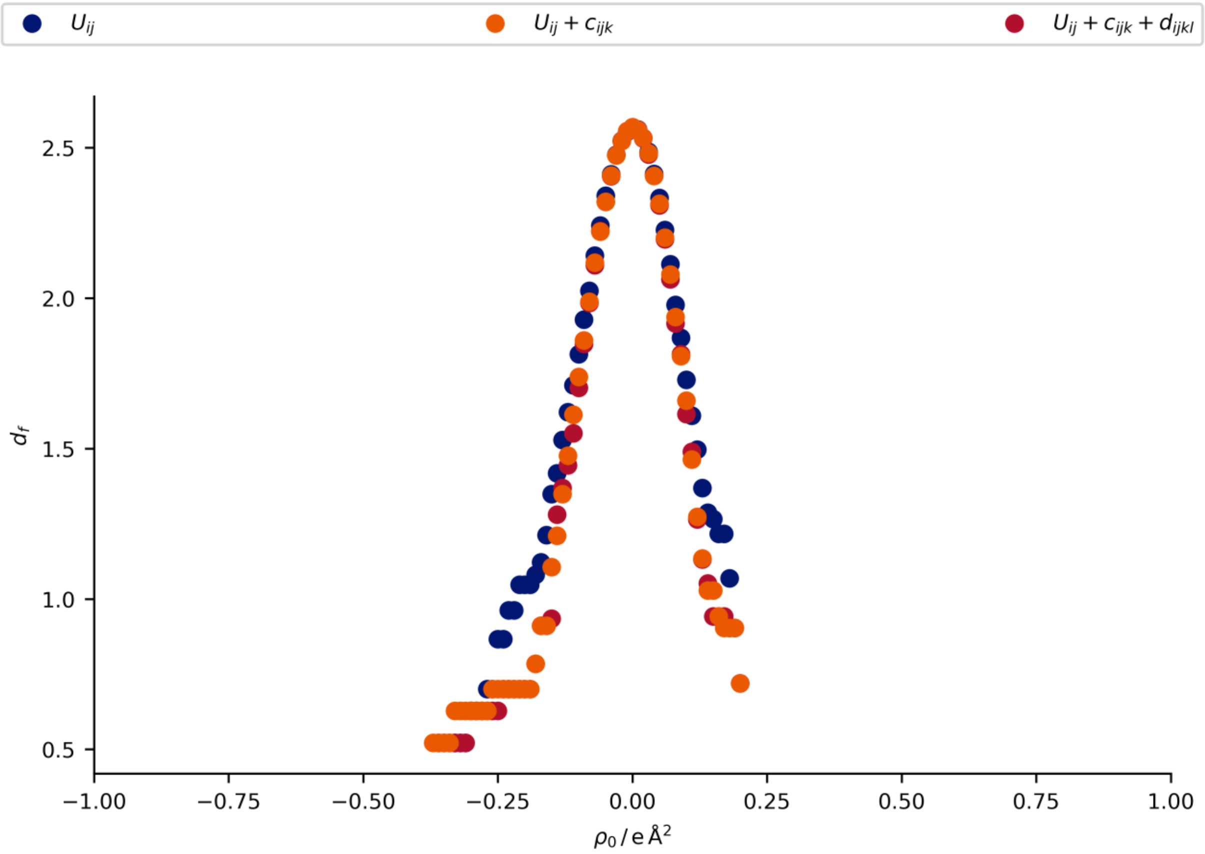

Figure S37 Henn-Meindl plots of the SCAN PAW calculations with Gram-Charlier refinement of the **Urea** dataset.

References

Adam Stash (2007). *DRKplot.* Moscow.

Dolomanov, O. V., Bourhis, L. J., Gildea, R. J., Howard, J. A. K. & Puschmann, H. (2009). *J. Appl. Cryst.* **42**, 339–341, doi:10.1107/S0021889808042726.

Kuhs, W. F. (1992). *Acta Cryst*. **A48**, 80–98, doi:10.1107/S0108767391009510.

Meindl, K. & Henn, J. (2008). *Acta Cryst.* **A64**, 404–418, doi:10.1107/S0108767308006879.

Midgley, L., Bourhis, L. J., Dolomanov, O., Peyerimhoff, N. & Puschmann, H. (20.11.2019). *Crystallographic Refinement using Non-Spherical Form Factors in olex2.refine*.

Monkhorst, H. J. & Pack, J. D. (1976). *Phys. Rev. B.* **13**, 5188–5192, doi:10.1103/PhysRevB.13.5188.

Sheldrick, G. M. (2015). *Acta Crystallogr.* **C71**, 3–8, doi:10.1107/S2053229614024218.

Verstraelen, T., Tecmer, P., Heidar-Zadeh, F., Boguslawski, K., Chan, M., Zhao, Y., Kim, T. D., Vandenbrande, S., Yang, D., González-Espinoza, C. E., Fias, S., Limacher, P. A., Berrocal, D., Malek, A. & Ayers, P. W. (2015). *HORTON 2.0.1*.

Volkov, A., Macchi, P., Farrugia, L. J., Gatti, C., Mallinson, P. R., Richter, T. & Koritsanszky, T. (2016). *XD2016*.

Wall, M. E. (2016). *IUCrJ*. **3**, 237–246, doi:10.1107/S2052252516006242.

Wieduwilt, E. K., Macetti, G. & Genoni, A. (2021). *J. Phys. Chem. Lett.* **12**, 463–471, doi:10.1021/acs.jpclett.0c03421.
